# Genome-wide profiling of Hfq-bound RNAs reveals the iron-responsive small RNA RusT in *Caulobacter crescentus*

**DOI:** 10.1128/mbio.03153-23

**Published:** 2024-03-21

**Authors:** Laura N. Vogt, Gaël Panis, Anna Schäpers, Nikolai Peschek, Michaela Huber, Kai Papenfort, Patrick H. Viollier, Kathrin S. Fröhlich

**Affiliations:** 1 Institute of Microbiology, Faculty of Biological Sciences, Friedrich Schiller University, Jena, Germany; 2 Department of Biology I, Microbiology, Ludwig-Maximilians-University Munich, Munich, Germany; 3 Department of Microbiology and Molecular Medicine, Faculty of Medicine/Centre Médical Universitaire, University of Geneva, Geneva, Switzerland; 4 Cluster of Excellence Balance of the Microverse, Friedrich Schiller University, Jena, Germany; The Pennsylvania State University, University Park, Pennsylvania, USA

**Keywords:** *Caulobacter*, small RNA, Hfq, iron starvation, NtrYX

## Abstract

The alphaproteobacterium *Caulobacter crescentus* thrives in oligotrophic environments and is able to optimally exploit minimal resources by entertaining an intricate network of gene expression control mechanisms. Numerous transcriptional activators and repressors have been reported to contribute to these processes, but only few studies have focused on regulation at the post-transcriptional level in *C. crescentus*. Small RNAs (sRNAs) are a prominent class of regulators of bacterial gene expression, and most sRNAs characterized today engage in direct base-pairing interactions to modulate the translation and/or stability of target mRNAs. In many cases, the ubiquitous RNA chaperone, Hfq, contributes to the establishment of RNA-RNA interactions. Although the deletion of the *hfq* gene is associated with a severe loss of fitness in *C. crescentus*, the RNA ligands of the chaperone have remained largely unexplored. Here we report on the identification of coding and non-coding transcripts associated with Hfq in *C. crescentus* and demonstrate Hfq-dependent post-transcriptional regulation in this organism. We show that the Hfq-bound sRNA RusT is transcriptionally controlled by the NtrYX two-component system and induced in response to iron starvation. By combining RusT pulse expression with whole-genome transcriptome analysis, we determine 16 candidate target transcripts that are deregulated, many of which encode outer membrane transporters. We hence suggest RusT to support remodeling of the *C. crescentus* cell surface when iron supplies are limited.

**IMPORTANCE:**

The conserved RNA-binding protein Hfq contributes significantly to the adaptation of bacteria to different environmental conditions. Hfq not only stabilizes associated sRNAs but also promotes inter-molecular base-pairing interactions with target transcripts. Hfq plays a pivotal role for growth and survival, controlling central metabolism and cell wall synthesis in the oligotroph *Caulobacter crescentus*. However, direct evidence for Hfq-dependent post-transcriptional regulation and potential oligotrophy in *C. crescentus* has been lacking. Here, we identified sRNAs and mRNAs associated with Hfq *in vivo*, and demonstrated the requirement of Hfq for sRNA-mediated regulation, particularly of outer membrane transporters in *C. crescentus*.

## INTRODUCTION

One essential hallmark of bacteria is their ability to effectively sense and react to fluctuations in the environment. Complex regulatory networks coordinate bacterial stress responses by converting external stimuli into defined changes in gene expression, and RNA-mediated control mechanisms efficiently complement protein-based regulation (1). Especially bacterial regulatory small RNAs (sRNAs) have consistently been linked to environmental responses and can critically impact gene expression control at the post-transcriptional level. Diverse in structure, size, and function, the most prevalent class of sRNAs commonly interact with complementary target messenger RNAs (mRNAs) through the formation of limited base-pairing interactions which results in changes in translation and/or stability of target transcripts (2). While sRNAs have the capacity to positively affect target gene expression, they most commonly act as repressors. When binding in the vicinity of the translation initiation region, sRNAs block ribosome recruitment. As a consequence, the sRNA:mRNA complex is usually rapidly turned over by ribonucleases (RNases) (3, 4). Alternatively, sRNAs can recruit RNases to the base-pairing site to facilitate transcript cleavage and decay without affecting translation initiation (5). Gene activation by sRNAs is based on target mRNA stabilization and stimulation of translation via base-pairing to alleviate self-inhibitory structures within the target, interfere with pre-mature transcription termination, or mask RNase recognition sites (6–9).

Bacterial riboregulators oftentimes require the assistance of RNA chaperones which stabilize sRNAs and facilitate annealing of target transcripts. The three major RNA chaperones currently known to foster sRNA activity in bacteria are the FinO family member ProQ, CsrA/Rsm, and Hfq, which belongs to the Lsm/Sm protein superfamily [reviewed in reference (10)]. Widely conserved in bacterial species, Hfq assembles in a homo-hexameric ring structure offering binding sites for single-stranded RNAs at both faces of the ring, the rim, and the intrinsically unstructured C-terminal domain which is appended to the core (11–13). The C-terminal domain has been resolved in a crystal structure of *Caulobacter crescentus* Hfq, revealing its ability to interact with positively charged residues on the rim to foster selective annealing of RNA (14).

In comparison to the well-understood standard models of bacterial RNA biology such as the Gram-negative enterobacteria *Escherichia coli* and *Salmonella* Typhimurium, studies on the role of Hfq in post-transcriptional control in *C. crescentus* have only scratched the surface (15). Most of the ~130 sRNA candidates previously identified by transcriptomic studies in *Caulobacter* still await further characterization, and a function of Hfq for the activity of the four sRNAs studied in more detail as yet has either not been observed (ChvR and GsrN) or not been addressed (CrfA and CcnA) (16–21).

A member of the highly diverse clade of *Alphaproteobacteria*, *C. crescentus* inhabits nutrient-poor freshwater environments and has evolved diverse mechanisms to adapt to severe nutrient fluctuations in this niche (22). The oligotrophic lifestyle of *C. crescentus* is for example reflected in the abundance of more than 60 genes encoding paralogs of outer membrane transporters of the family of TonB-dependent transporters (TBDRs). TBDRs catalyze the specific uptake of nutrients from the dilute environment including substrates like carbohydrates, vitamin B12, and iron-scavenging siderophores (23–27). Iron serves as an essential cofactor in central metabolism and respiration, yet due to its poor solubility in the abundant, ferric form (Fe^3+^), it is a limiting nutrient in most biological systems (28). On the other hand, free intracellular ferric iron can damage biomolecules through reactive oxygen species, which eventually causes cell death (29). Iron homeostasis is thus finely controlled in bacteria, and oftentimes involves a transcriptional master regulator termed Fur (ferric uptake regulator) which, when in complex with iron, binds so-called Fur boxes predominantly in promoters to repress expression of genes controlling iron uptake (30). In *E. coli* and related enterobacteria, the Fur regulon includes an Hfq-dependent sRNA, RyhB, which serves as the negative arm of the regulatory circuit and represses the synthesis of non-essential iron-binding proteins to increase the availability of iron (31). While Fur is also the master transcriptional regulator of iron homeostasis in *C. crescentus* (32), an sRNA modulating gene expression at the post-transcriptional level in response to iron depletion remains to be identified.

Here, we conducted RNA co-immunoprecipitation experiments to catalogue direct ligands of Hfq in *C. crescentus*. Our results reveal that of all sRNAs expressed in the early stationary phase, only a subset is associated with Hfq in this bacterium, including sRNA CCNA_R0199. We found that the transcript is specifically induced in response to high levels of zinc and iron starvation, and renamed it RusT (RNA upregulated in iron starvation). Forward genetics in combination with biochemical analyses unveiled that transcription of RusT is under the control of NtrYX, a conserved two-component system (TCS) recently implicated in cell envelope regulation in alphaproteobacteria. Moreover, transcriptome analyses revealed that RusT, together with Hfq, post-transcriptionally controls at least 16 transcripts, including seven membrane transporters. In summary, our study presents evidence for Hfq-dependent post-transcriptional regulation through an sRNA in *C. crescentus*, confirming that the chaperone is not only mediating RNA stability but also functions as an important mediator of RNA-based post-transcriptional control of the membrane proteome in this oligotroph.

## RESULTS

### Profiling of Hfq-associated RNA in *C. crescentus*

**Fig 1:**
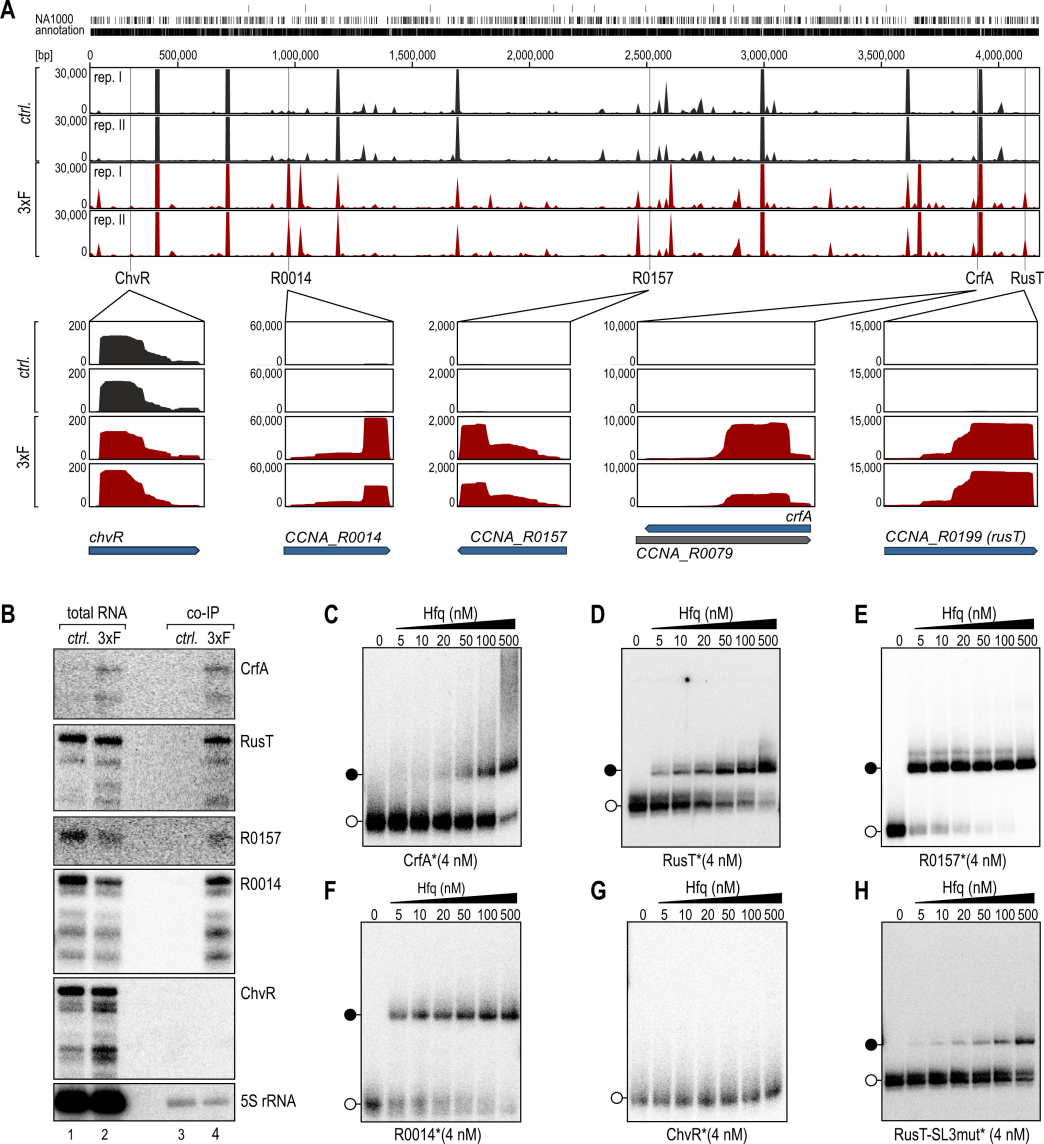
Specific association of RNA with the Hfq protein in *C. crescentus*. (**A**) *C. crescentus* NA1000 carrying a chromosomally encoded *3xFLAG-hfq* allele was cultured in complex PYE medium to an OD_660_ of 1.0, an untagged wild-type strain served as control. Following the co-immunoprecipitation of RNA-Hfq complexes from cell lysates using a monoclonal antibody directed against the FLAG-tag, isolated RNA was converted into cDNA, and subjected to high-throughput sequencing analysis. cDNA reads of the control (*ctrl*.; black) and 3xFLAG-Hfq co-IP (3xF; red) were mapped to the *C. crescentus* NA1000 genome (two replicates each, rep. I and rep. II). Examples of enrichment patterns of five sRNAs are shown as enlarged panels below the sequencing tracks. For each plot, a scale indicates the number of mapped reads per position. (**B**) Northern blot analysis of total RNA samples and upon co-IP of *C. crescentus* wild-type (*ctrl*.) and cells expressing 3xFLAG-Hfq (3xF) to determine recovery profiles for five selected sRNAs. 5S rRNA served as loading control. (**C–H**) Native gel electrophoretic mobility shift assay (EMSA) using *in vitro*-synthesized, 5′ end-labeled sRNAs (**C**) CrfA, (**D**) RusT, (**E**) R0157, (**F**) R0014, (**G**) ChvR, or (**H**) RusT-SL3mut (all 4 nM) with increasing concentrations of purified *C. crescentus* Hfq protein as indicated. Open circles indicate free sRNAs, black circles indicate sRNA-Hfq complexes.

A previous study reporting on the purification of RNA crosslinked to Hfq via UV illumination identified nine potential sRNA ligands in *C. crescentus* (15), but we assumed that the Hfq interactome was likely to be more extensive. To close this gap, we performed co-immunoprecipitation (co-IP) of RNA with a functional Flag-tagged derivative of Hfq (Fig. S1). Setting a cutoff at ≥3-fold enrichment in the co-IP sample, we detected 311 transcripts bound by Hfq, including 290 mRNAs, 19 sRNAs, and 2 rRNAs (Table S1; Fig. 1A).

The fraction of sRNAs purified from the co-IP sample more than doubled relative to the control (Fig. S2A), and we listed the candidates based on their enrichment (Table 1). We verified the results of the RNA sequencing experiment for four sRNAs with the highest enrichment (CrfA, RusT, R0157, and R0014) using Northern blot analysis (Fig. 1B), and included ChvR as Hfq-independent control sRNA (14, 17). As expected, ChvR was not recovered with Hfq, while CrfA, RusT, R0157, and R0014 were detected in the co-IP sample. However, we observed that expression of CrfA, an sRNA induced during starvation (18), was increased in the total RNA input sample of the *3xFLAG::hfq* strain when compared to the control strain (Fig. 1B; Fig. S3), resulting in a potential overestimation of the relative enrichment of this RNA. All other tested sRNAs had comparable expression patterns in both strains (Fig. S3). To further corroborate our results, we performed native gel electrophoretic mobility shift assays (EMSAs) using purified *C. crescentus* Hfq protein and *in vitro* transcribed sRNAs. As expected, Hfq formed stable complexes with CrfA, RusT, R0157, and R0014 (Fig. 1C through F), but not with ChvR (Fig. 1G).

**TABLE 1:** Hfq-associated sRNAs (≥3-fold enrichment)

Gene	Enrichment	FDR-adj. *P* value
*CCNA_R0093 (crfA)*	172.1	9.5 E−80
*CCNA_R0199 (rusT)*	73.8	5.8 E−150
*CCNA_R0157*	63.8	1.8 E−111
*CCNA_R0014*	51.7	3.4 E−53
*CCNA_R0188*	49.2	1.4 E−120
*CCNA_R0143*	34.9	8.2 E−32
*CCNA_R0097*	20.7	1.9 E−63
*CCNA_R0195*	17.6	1.7 E−63
*CCNA_R0040*	11.4	1.9 E−40
*CCNA_R0133*	9.1	1.0 E−41
*CCNA_R0025*	8.1	1.2 E−29
*CCNA_R0127*	7.0	5.4 E−44
*CCNA_R0098*	5.7	1.0 E−18
*CCNA_R0156*	5.6	3.1 E−14
*CCNA_R0016*	5.0	1.3 E−15
*CCNA_R0184*	4.3	2.3 E−16
*CCNA_R0175*	3.4	3.6 E−15
*CCNA_R0117*	3.3	4.5 E−11

### RusT is a conserved sRNA interacting with Hfq

**Fig 2:**
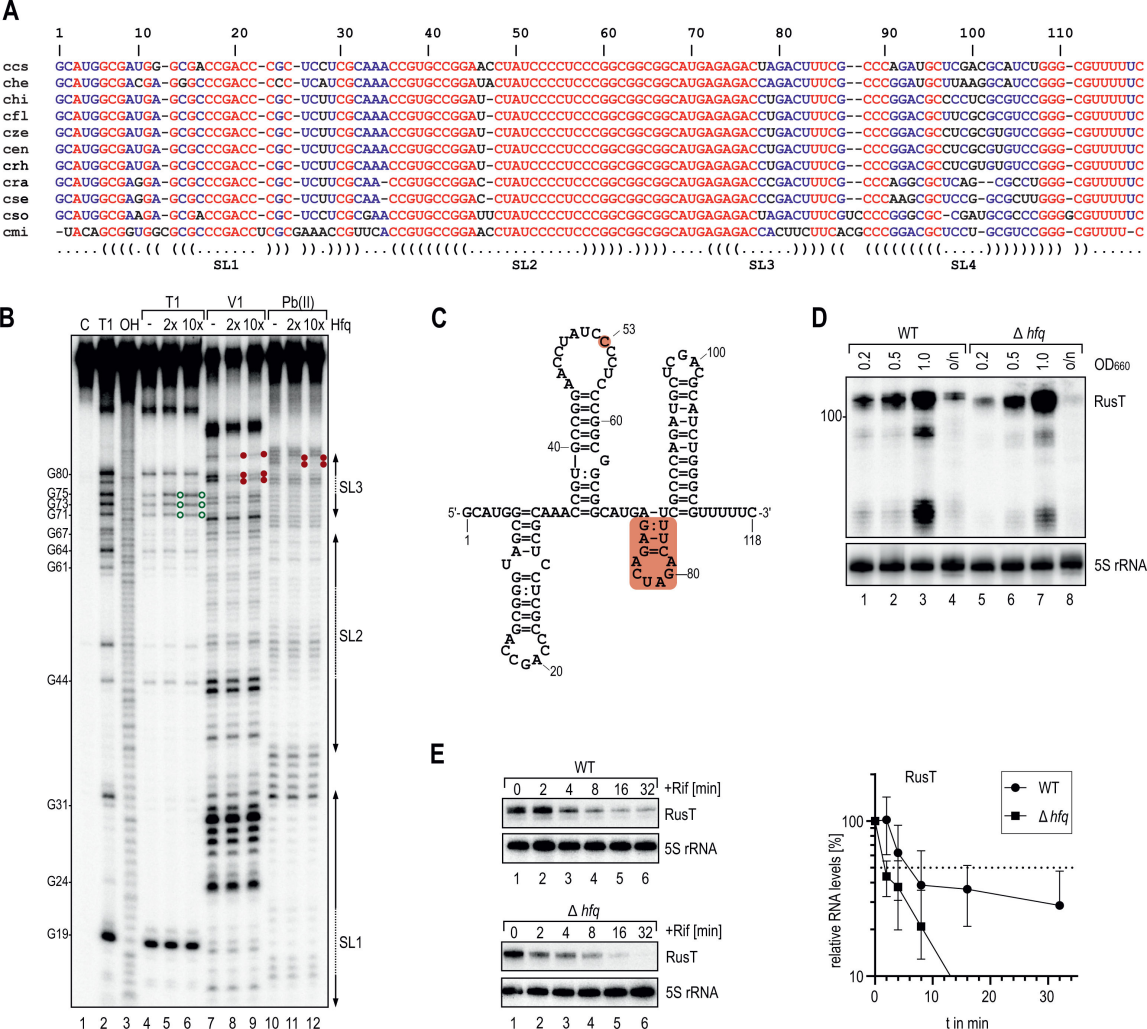
Conservation, structure, and Hfq-dependence of RusT. (**A**) Non-redundant alignment of the RusT sRNA with orthologous sRNAs of the genus *Caulobacter* (ccs: *C. crescentus* NA1000; che: *C. henricii* CB4; chi: *C. hibisci* strain KACC 18849; cfl: *C. flavus* strain RHGG3; cze: *C. zeae* strain 410 SGCZ15; cen: *C. endophyticus* strain 774; crh: *C. rhizosphaerae* strain KCTC 52515; cra: *C. radicis* strain 695; cse: *C. segnis* strain TK0059; cso: *C. soli* strain Ji-3-8; cmi: *C. mirabilis* strain FWC 38). Colors indicate full (red); partial (blue) or no (black) conservation. The predicted secondary structure of *C. crescentus* RusT is indicated below the alignment. (**B**) *In vitro* structure probing of 5′ end-labeled RusT sRNA (0.4 pmol) with RNase T1 (lanes 4–6), RNase V1 (lanes 7–9), and lead(II) (lanes 10–12) in the presence and absence of purified Hfq protein (0, 0.8 pmol or 4 pmol, respectively). RNase T1 and alkaline (OH) ladders of RusT were used to map cleavage fragments, and positions of mapped G-residues are marked relative to the TSS. Increased cleavage is marked with green open circles, reduced cleavage is marked with red filled circles. (**C**) Secondary structure of RusT as determined by chemical probing shown in (**B**). The sRNA region interacting with Hfq (nt 73–84) and residue C53 are highlighted in red. (**D**) Northern blot analysis of RusT expression in *C. crescentus* wild-type and an isogenic *hfq* mutant strain. RNA was collected at the indicated optical density (OD_660_) or after 24 h of growth (o/n) in PYE medium. 5S rRNA served as loading control. (**E**) Determination of RusT stability. Northern blot analysis of RNA obtained from wild-type or Δ*hfq* cells grown in minimal M2G medium to exponential phase (OD_660_ of 0.5). Total RNA samples were collected at indicated time points prior to, and after addition of rifampicin to inhibit transcription. The time point at which 50% of RusT had been decayed (dashed line) was calculated to determine RNA half-life. Error bars indicate the standard deviation of three independent biological replicates.

We selected the previously uncharacterized RusT sRNA (annotated as CCNA_R0199) which was strongly enriched in our co-IP experiment (>70-fold; Table 1), to study the functions of an Hfq-associated sRNA in *C. crescentus*. RusT is transcribed as a 118-nt long RNA from the plus-strand of the intergenic region between *CCNA_03820* (encoding an outer membrane lipoprotein carrier protein; *lolA*) and *CCNA_03821* (encoding exodeoxy-ribonuclease III involved in DNA repair; *xth*). Homologs of *rusT* with little sequence variation are encoded at this genomic locus in other *Caulobacteraceae* closely related to *C. crescentus* (Fig. S4; Fig. 2A). To determine the secondary structure of RusT in the presence and absence of Hfq we employed chemical probing. To this end, we incubated *in vitro* transcribed, 5′ end-labeled RusT sRNA with increasing concentrations of purified Hfq protein, and subsequently subjected the RNA to partial digestion with RNase T1 (preferentially cleaving after single-stranded guanine residues), RNase V1 (preferentially cleaving double-stranded regions), and lead(II) acetate (preferentially cleaving single-stranded regions). The resulting digestion pattern (Fig. 2B) revealed that RusT contains four stem-loops, of which the last one is followed by a polyU-stretch likely functioning as a Rho-independent termination signal (Fig. 2C). The addition of Hfq caused a change in the cleavage pattern between nt 72 and 87 (corresponding to stem-loop 3), suggesting that the RNA-binding protein alters the secondary structure of the sRNA when occupying this site. Indeed, when we used a mutant of RusT lacking the third stem-loop (RusT-SL3mut) in EMSAs with Hfq, we observed a strong decrease in the binding affinity compared to wild-type RusT (Fig. 1D and H).

To test if Hfq protects RusT from degradation, we compared the RusT levels in wild-type and *hfq* mutant strains (Fig. 2D) and observed equal or even elevated levels (depending on the time point of sampling) of RusT in the absence of Hfq. To determine whether this result was due to either higher transcription rates or increased stability of RusT in the mutant, we first determined the half-life of the sRNA. Both wild-type and *hfq* mutant cells were grown to exponential phase, and RNA decay was monitored after inhibition of transcription with rifampicin (Fig. 2E). RusT stability decreased more than threefold in the absence of Hfq (Fig. 2E), as frequently observed for Hfq-associated sRNAs in other species (6, 33, 34). Similarly, decay of the Hfq-associated sRNA R0014 was accelerated in the *hfq* mutant strain, while half-life of the Hfq-independent ChvR sRNA was not affected (Fig. S5A and B). Since RusT stability was decreased in the absence of Hfq, the increase of RusT steady-state levels in the *hfq* mutant strain was likely a result of increased sRNA transcription. Indeed, when we expressed *rusT* under control of the unrelated, vanillate-inducible promoter (pP_van_-RusT), sRNA levels in the absence of Hfq were no longer augmented (Fig. S5C).

### Iron starvation induces RusT transcription

**Fig 3:**
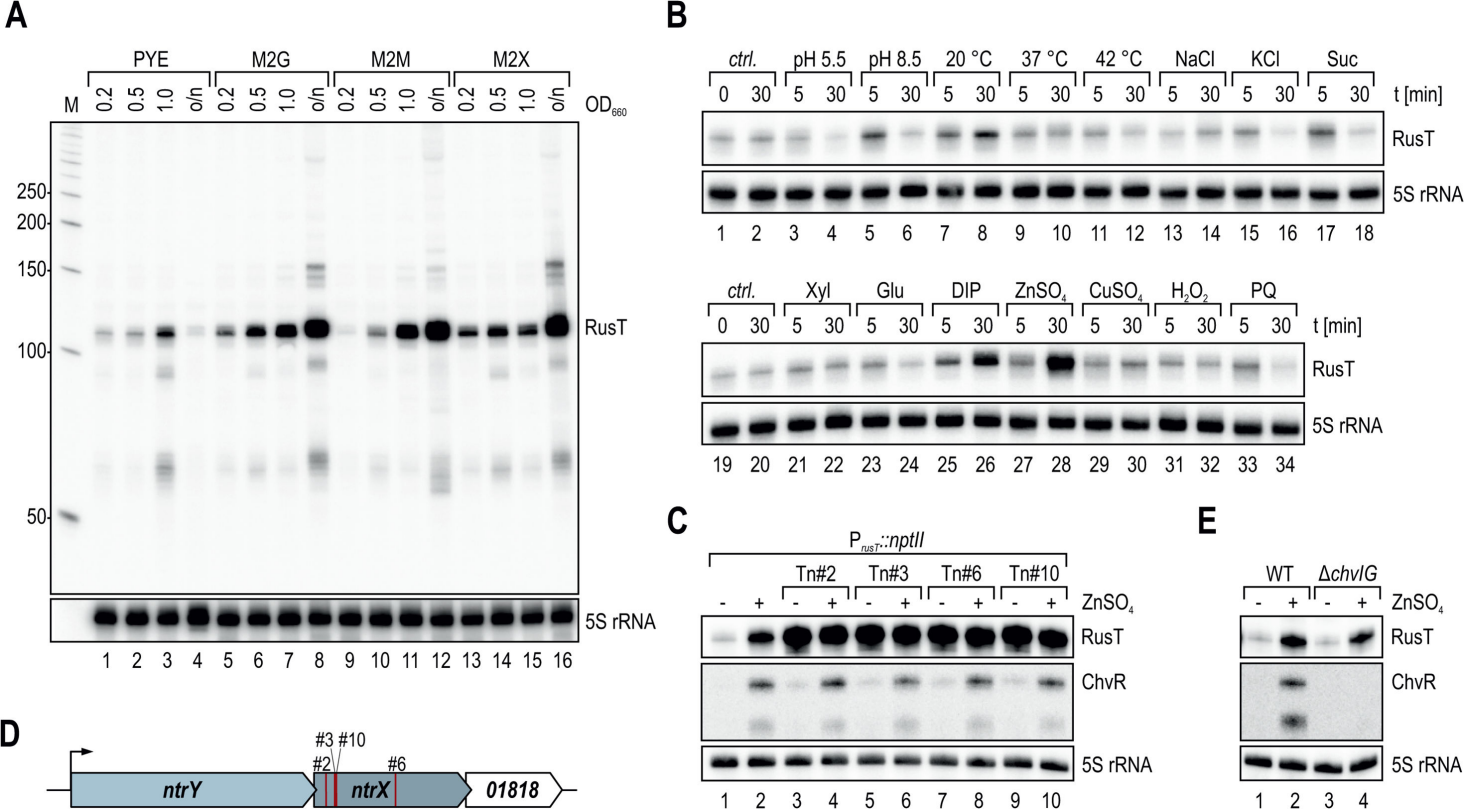
RusT is induced in response to iron starvation. (**A**) Northern blot analysis of RusT expression in PYE or minimal M2 medium supplemented with glucose (M2G), maltose (M2M), or xylose (M2X) as carbon source. RNA was collected at the indicated optical density (OD_660_) or after 24 h of growth (o/n). 5S rRNA served as loading control. (**B**) Total RNA was prepared from cells grown in PYE to OD_660_ of 0.4 at different time points without (*ctrl*.) or upon treatment (5 min and 30 min) of the culture with the indicated stresses (pH 5.5: PYE at pH 5.5; pH 8.5: PYE at pH 8.5; 20 °C/37 °C/42°C: growth at alternative temperature; NaCl/KCl/Suc: osmotic stress at 85 mM NaCl/40 mM KCl/150 mM sucrose, respectively; Xyl: addition of xylose [f.c. 0.2%]; Glu: addition of glucose [f.c. 0.2%]; DIP: addition of 2,2-dipyridyl [f.c. 200 µM]; ZnSO_4_: addition of ZnSO_4_ [f.c. 75 µM]; CuSO_4_: addition of CuSO_4_ [f.c. 30 µM]; H_2_O_2_/PQ: oxidative stress at 10 mM hydrogen peroxide or paraquat, respectively). RusT expression in response to stress was determined by Northern blot analysis; 5S rRNA served as loading control. (**C**) Northern Blot analysis of RusT and ChvR expression in *C. crescentus* carrying the transcriptional reporter P*_rusT_::nptII*, without and selected clones with insertion of a mini-*himar1* transposon (Mar2xT7). Total RNA samples were prepared from cells at OD_660_ of 0.5 in PYE prior to and 30 min after addition of 75 µM ZnSO_4_. 5S rRNA served as loading control. (**D**) Mapped positions of transposon insertions in *ntrX* of the clones analyzed in (**C**) are indicated in red. (**E**) Expression of sRNAs RusT and ChvR was analyzed on Northern blots using RNA samples prepared from *C. crescentus* wild-type and a Δ*chvIG-hprK* mutant strain grown as described in (**C**).

To probe the transcriptional regulation of RusT in *C. crescentus*, we next determined RusT abundance by Northern blot analysis of total RNA samples collected from cells grown in complex (PYE) and defined media (M2 supplemented with either glucose, maltose, or xylose as sole carbon source), and after stress induction under 16 different conditions (Fig. 3A and B). RusT levels were elevated in cells cultivated in minimal medium and most abundant in samples collected after overnight growth (Fig. 3A). However, when exponentially growing cells were treated with either the chelator 2,2-dipyridyl (DIP) or 75 µM of ZnSO_4_, respectively (Fig. 3B, lanes 26 and 28), a ~5-fold and ~9-fold increase in RusT levels was observed. Addition of DIP is known to induce iron starvation (31), and similarly, excess of zinc results in perturbation of the iron homeostasis, leading to potential mismetallation of iron-dependent enzymes (35–37). In many bacteria, iron starvation and concomitant de-repression of the Fur regulon also induces sRNAs including RyhB in the enterobacteria or PrrF, NrrF, and FsrA sRNAs of *Pseudomonas aeruginosa*, *Neisseria meningitidis*, and *Bacillus subtilis*, respectively (38–40). However, contrary to the abovementioned examples, we suspected that *rusT* transcription was not directly repressed by Fur as we were unable to identify the well-defined consensus binding site within the promoter P*_rusT_* (32), suggesting that another regulator controls the expression of the sRNA.

**Fig 4:**
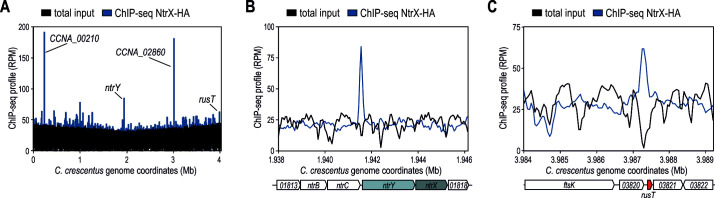
Determination of NtrX binding sites on the genome of *C. crescentus*. (**A**) Genome-wide profiles of reads for NtrX-HA ChIP-Seq (blue) and total input control (black). Selected peaks are assigned, a list of all significantly enriched sites is provided in Table S2. (**B, C**) Traces of NtrX-HA ChIP-Seq (blue) and total input control (black) upstream of the *ntrXY-CCNA_01818* operon (**B**) or at the *rusT* promoter (**C**).

To identify regulators of *rusT* transcription, we integrated a transcriptional reporter into the *Caulobacter* chromosome in which expression of the *nptII* gene (conferring resistance to kanamycin) is driven by P*_rusT_*. We capitalized on the low activity of P*_rusT_* and the resulting inability of the reporter strain to grow on plates supplemented with high concentrations of kanamycin ( 20 µg/mL; Kan20). Upon mutagenesis with the mini-*himar1* transposon (Mar2xT7, conferring resistance to gentamycin), we selected clones growing on Kan20 and gentamycin. When testing RusT levels in response to zinc for 10 individual clones, we identified four mutants in which the sRNA was constitutively expressed independent of the presence of the inducer (Fig. 3C). The *himar1* insertion site in all four mutants was mapped to *ntrX*, encoding the response regulator of the conserved NtrYX TCS (Fig. 3D). Originally, the NtrYX system has been associated with the regulation of nitrogen metabolism including denitrification, nitrogen assimilation, and fixation in diverse bacteria (41–45), but has also been implicated to respond to iron starvation of *Paracoccus denitrificans* (46). To probe for a direct involvement of NtrX in RusT transcription, we performed chromatin immunoprecipitation coupled to deep sequencing (ChIP-Seq) in a *C. crescentus* strain harboring an HA-tagged version of *ntrX* (*ntrX::HA*; (47)) in lieu of the wild-type version. We detected 23 peaks that were significantly enriched (fold-enrichment ≥2) over the background, including a site upstream of the *ntrYX* transcriptional start site (TSS) (Fig. 4A and B; Table S2). Potential autoregulation has likewise been suggested for the NtrYX system of *Rhodobacter sphaeroides* where NtrX also binds its own promoter (45). In addition, the ChIP-Seq analysis revealed efficient occupancy of P*_rusT_* by NtrX (Fig. 4C), indeed suggesting that the response regulator is directly involved in transcriptional control of RusT and functioning as a repressor of sRNA expression under non-inducing conditions.

### Identification of RusT target genes

**Fig 5:**
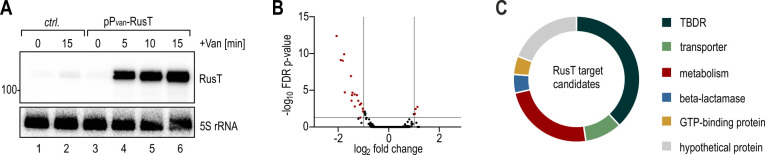
Pulse-overexpression of RusT reveals target genes of the sRNA. (**A**) *C. crescentus* Δ*vanAB* carrying either an empty control vector (pBV-MCS6; *ctrl*.) or the expression plasmid pP_van_-RusT were grown in M2G to an OD_660_ of 0.8. Total RNA was prepared from cells collected prior to and at indicated time points after addition of vanillate. Expression of RusT and 5S rRNA (loading control) was determined by Northern blot analysis. (**B**) Volcano-plot analysis of gene expression changes in response to pulse-induction (15 min) of RusT. Genes with fold changes ≥2 and an FDR-adj. *P* value of ≤0.05 were considered as significantly different and are highlighted in red. (**C**) Classification of RusT target candidates based on annotated gene functions.

While no Hfq-dependent activity of a *trans*-acting sRNA is currently known for *C. crescentus* (48, 49), we were curious to determine the physiological function of RusT. To identify potential direct targets of the sRNA, we globally assessed transcriptome differences in response to pulse-overexpression of RusT. To this end, we cultivated wild-type cells carrying either an empty control vector or the vanillate-inducible pP_van_-RusT in M2G (OD_660_ of 0.8), and isolated total RNA samples before and after addition of vanillate. Under this condition, RusT is only weakly expressed, and competition with the endogenous sRNA is not relevant to the experiment. Compared to the control, RusT levels increased ~13-fold within 15 min in the presence of the inducer (Fig. 5A; Fig. S6). We next converted the total RNA samples into cDNA which we subjected to high-throughput sequencing (HTPS) analysis. In total, 23 transcripts met our cutoff criteria for differentially expressed genes (≥10 reads; ≥2-fold regulation; FDR-adj. *P* value ≤ 0.05), of which four were upregulated and 19 were downregulated in the presence of RusT (Table 2; Fig. 5B). Not surprisingly, RusT sRNA levels were strongly induced. In addition, also CrfA sRNA increased ~2-fold, a result which is likely an artifact of the pulse-overexpression due to increased Hfq occupancy by RusT upon the addition of vanillate to the culture (Fig. S6). Two additional transcripts were more abundant in response to RusT expression when compared to the control, *CCNA_03360* (encoding tagatose-1,6-bisphosphate aldolase; 2-fold upregulation) as well as *CCNA_01820* (2.1-fold upregulation), encoding *hflX*, the second gene in the *hfq-hflX* operon [the *hfq* (*CCNA_01819*) mRNA increased 1.98-fold in our experiment, and was excluded by our cutoff criteria]. The large set of repressed genes included transcripts encoding TBDRs (*CCNA_02895*, *CCNA_03263*, *CCNA_01042*, *CCNA_01738*, *CCNA_00210*, *CCNA_03248*, *CCNA_00338*, and *CCNA_00573*), other types of transporters [major facilitator superfamily (MFS) transporter *CCNA_02400*, porin *CCNA_01475* (*ompW*)], various metabolic factors [*CCNA_02380*, *CCNA_03181*, *CCNA_03774*, and *CCNA_02047* (*glnA*)], a beta-lactamase family protein (*CCNA_03294*) as well as several hypothetical proteins (*CCNA_01043*, *CCNA_02741*, *CCNA_02342*, and *CCNA_02379*). When we analyzed the list of potential RusT targets we realized that a significant portion had been previously identified to be down-regulated in response to iron starvation and/or in strains deficient for Fur (Table 2; (50)), corroborating our finding that RusT is activated when iron homeostasis is disturbed (Fig. 3B).

**TABLE 2:** Differentially expressed transcripts in response to RusT pulse-expression (≥2-fold change)

Gene ID	Gene product	Fold change	FDR-adj. *P* value	*gfp* fusion (relative to TSS)	Downregulation in low iron conditions^*a*^
CCNA_02895	TonB-dependent receptor	−4.2	4.1 E−13	−269 to +60	Yes
CCNA_03263	TonB-dependent receptor	−3.8	7.7 E−10	−116 to +60	Yes
CCNA_01042	TonB-dependent receptor	−3.5	9.5 E-10	−225 to +60	Yes
CCNA_02400	Transporter, major facilitator superfamily	−3.4	1.2 E−10	−32 to +60	
CCNA_01475	OmpW family outer membrane protein	−3.3	1.9 E−05	−40 to +75	Yes
CCNA_01738	TonB-dependent outer membrane receptor	−2.8	1.2 E−07	−86 to +60	
CCNA_00210	TonB-dependent receptor	−2.8	2.6 E−4	−59 to +60	Yes
CCNA_02380	Aspartate 1-decarboxylase	−2.8	4.6 E−05	−71 to +60	
CCNA_03181	Alcohol dehydrogenase	−2.7	2.1 E−05	−156 to +60	
CCNA_01043	Hypothetical protein	−2.6	3.6 E−05	−43 to +60	
CCNA_03248	TonB-dependent receptor	−2.5	1.6 E−03	−49 to +60	
CCNA_02741	Conserved hypothetical protein	−2.4	4.6 E−05	−99 to +75	
CCNA_03774	Citrate lyase beta chain/citryl-CoA lyase subunit	−2.3	2.7 E−04	−29 to +60	Yes
CCNA_00338	TonB-dependent receptor	−2.2	9.2 E−04	−94 to +60	
CCNA_02047	Glutamine synthetase GlnA	−2.2	0.05	−424 to +60; −42 to +60	
CCNA_00573	TonB-dependent receptor	−2.1	6.4 E−04	No tss	
CCNA_02342	Hypothetical protein with pentapeptide repeats	−2.1	6.4 E−04	−35 to +60	
CCNA_03294	Beta-lactamase family protein	−2.0	3.3 E−03	−96 to +60	
CCNA_02379	Hypothetical protein	−2.0	7.7 E−04	−71 to +60	
CCNA_03360	Tagatose-1,6-bisphosphate aldolase	2.0	0.02	−282 to +60	
CCNA_01820	GTP-binding protein	2.1	3.6 E−03	−179 to +60; −53 to +60	
CCNA_R0093	Non-coding RNA CrfA	2.2	1.8 E−03		
CCNA_R0199	Non-coding RNA RusT	72.4	5.6 E−50		

^*a*^ As determined in reference (50).

### Reporter fusions confirm post-transcriptional regulation by RusT

To investigate the potential post-transcriptional regulation of the newly identified RusT target candidates, we designed 21 translational fusions to the green fluorescent protein (*gfp*) under control of the constitutive *rsaA* promoter (17). Base-pairing sRNAs typically interact with their target transcripts at sites located within the 5′ UTR or the early coding sequence of the mRNA (51). We fused the sequence from the TSS up to the first 15–20 codons of each target gene to the second codon of *gfp* (Table 2; Fig. S7). For genes organized in operons, we made a fusion to the first gene of the polycistronic mRNA. We omitted target candidate *CCNA_00573* from our analysis as we and others were unable to detect the TSS of the corresponding mRNA (17, 52).

**Fig 6:**
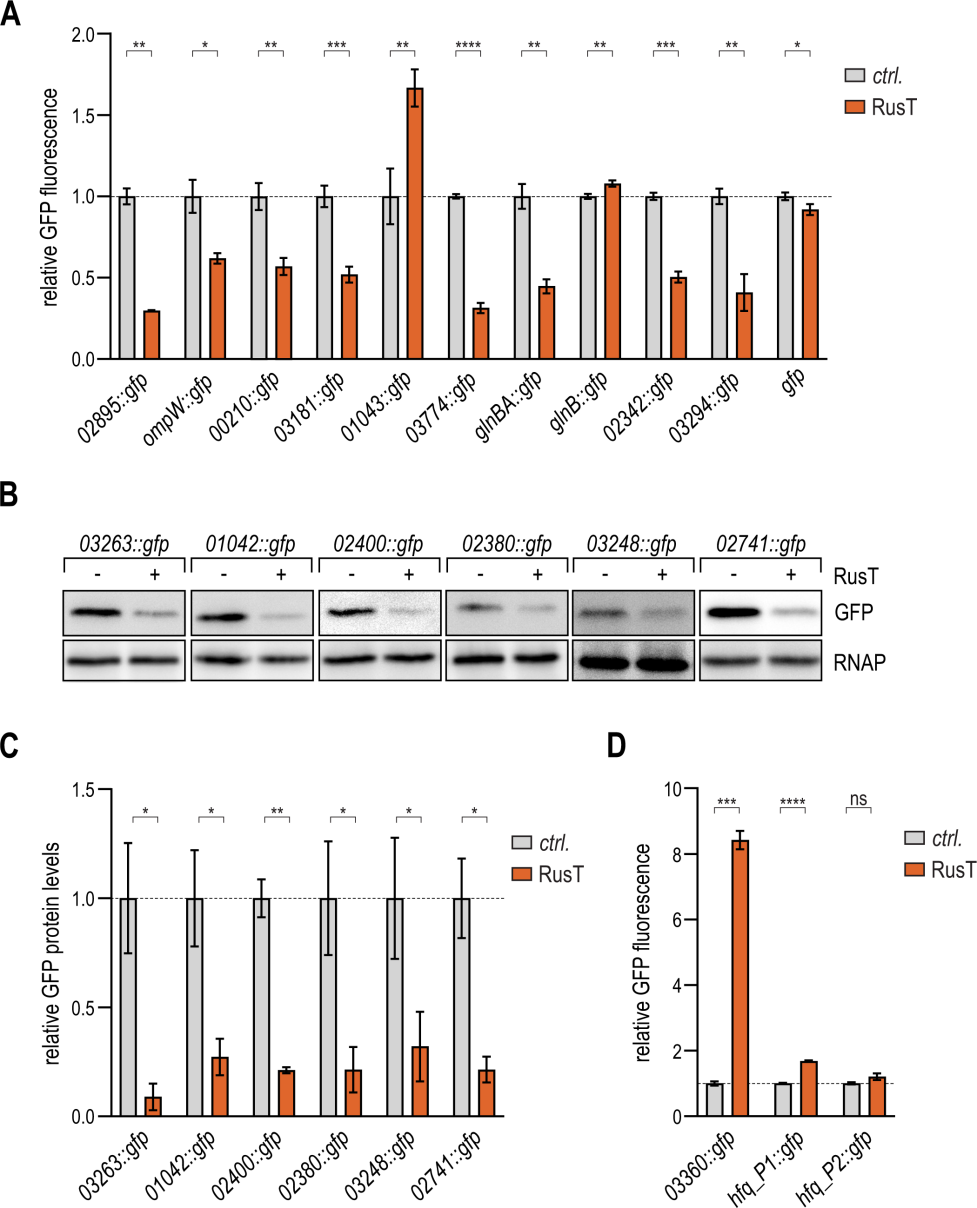
Regulation of RusT target reporter fusions. (**A–D**) Regulation of target reporter fusions by RusT. *C. crescentus* Δ*vanAB* Δ*rusT* cells carrying the indicated *gfp* reporter fusion in combination with either an empty control vector (pBV-MCS6; *ctrl*.), or the expression plasmid pP_van_-RusT were grown overnight in the presence of vanillate, and GFP expression was quantified either by fluorescence intensity measurements (**A, D**) or by Western blot analysis of total protein samples using RNA polymerase (RNAP) as loading control [B; quantification in (**C**)]. For the quantification of each GFP-fusion, expression levels in the presence of the control plasmid were set to 1, and relative changes were determined for cells expressing RusT. GFP levels were calculated from three biological replicates; error bars indicate the standard deviation.

We integrated the *gfp* reporter fusions in a *C. crescentus* Δ*vanAB* Δ*rusT* mutant carrying either an empty control plasmid, or the construct pP_van_-RusT to overexpress RusT. Cells were harvested upon overnight growth in PYE (supplemented with the inducer vanillate), and GFP expression was quantified by fluorescence intensity measurements (Fig. 6A and D). Fluorescence was reduced in the presence of RusT for eight target fusions (*02895::gfp*, *ompW::gfp, 00210::gfp, 03181::gfp*, *03774::gfp*, *glnBA::gfp, 02342::gfp,* and *03294::gfp*), while sRNA overexpression did not affect GFP production of a *gfp* control construct. An additional six fusions (*03263::gfp*, *01042::gfp*, *02400::gfp*, *02380::gfp*, *03248::gfp*, and *02741::gfp*) were validated as negatively regulated by RusT through immunoblot analysis using an anti-GFP antibody since the fluorescence of these reporters was below the detection limit (Fig. 6B and C). No negative regulation of *gfp* fluorescence in the presence of RusT was observed for two fusions, *glnB::gfp* and *01043::gfp*. In both cases, however, the two target candidates encode short leader peptides for the *glnBA* and *CCNA_01043-01042* operons, respectively. The longer reporter fusions comprising the second genes of these operonic structures are both regulated by RusT (Fig. 6A through C). We thus assume that target repression requires a regulatory element located downstream of the leader sequence. Reporter fusions *01738::gfp* and *00338::gfp* were excluded from the analysis as we were unable to detect GFP production for these constructs (not shown).

Our transcriptome analysis also revealed a positive effect of RusT expression on two target candidates (Table 2; Fig. 5B), and we confirmed upregulation of the *03360::gfp* fusion in the presence of RusT (Fig. 6D). As expression of the *hfq-hflX* operon is controlled through two promoter elements P1 and P2 (17, 52), we constructed two reporter variants (Fig. S7U and V). An increase in GFP fluorescence upon RusT overexpression was solely observed for the reporter fusion harboring the longer 5′ UTR (*hfq_P1::gfp*; Fig. 6D). Since input through the second, proximal promoter P2 included in this fusion could not be excluded, we also performed primer extension analysis of the native *hfq-hflX* transcripts. Pulse expression of RusT resulted in an accumulation of the shorter but not the longer transcript (Fig. S8). Taken together, these results point toward an increase of transcriptional activity from the second, proximal promoter P2 in response to RusT overexpression; however, the exact mechanism of regulation awaits further characterization.

### RusT function requires the aid of Hfq

**Fig 7:**
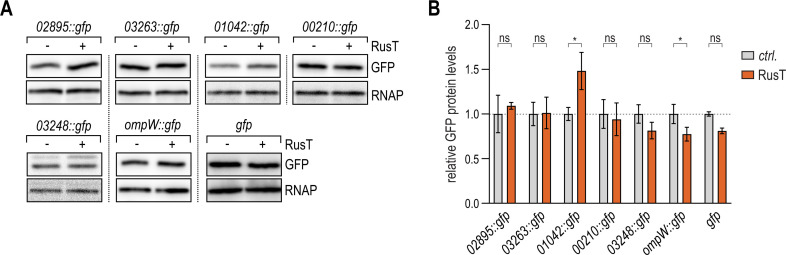
Target regulation by RusT is dependent on Hfq. (**A, B**) Regulation of target reporter fusions by RusT in the absence of Hfq. *C. crescentus* Δ*vanAB* Δ*rusT* Δ*hfq* cells carrying the indicated *gfp* reporter fusion in combination with either an empty control vector (pBV-MCS6; *ctrl*.), or the expression plasmid pP_van_-RusT were grown overnight in the presence of vanillate. Total protein samples were analyzed on Western blots to determine GFP expression; RNAP served as loading control. (**B**) Quantification of GFP expression as described in (**A**); error bars indicate the standard deviation from three biological replicates.

Tight control of outer membrane proteins is critical for cell survival, and many proteins involved in substrate transport across the outer membrane are controlled through the activity of sRNAs in enterobacteria (53, 54). Likewise, TBDRs spanning the outer membrane represented the largest class of confirmed RusT targets in *Caulobacter*, suggesting that the sRNA is involved in remodeling the cell envelope in response to iron deficiency. Different from many other Hfq-associated sRNAs, RusT was also well expressed in an *hfq* mutant background (Fig. 2D). We therefore sought to determine whether Hfq was at all required for RusT-dependent regulation. To this end, we selected the top five deregulated candidates encoding TBDRs (*02895::gfp*, *03263::gfp*, *01042::gfp*, *00210::gfp*, and *03248::gfp*) and *ompW::gfp* for further analysis, and transferred our reporter system into Δ*vanAB* Δ*rusT* Δ*hfq* cells. We again determined GFP levels for each of the six fusions in the presence or absence of RusT by immunoblot analysis using antibodies to GFP. Although we confirmed that overexpression from the P_van_ promoter resulted in similar sRNA levels when compared to the wild-type background (Fig. S9), RusT-dependent downregulation of the reporter fusions was abrogated in all cases (Fig. 7A and B). Collectively, our results suggest that the binding of Hfq not only increases the stability of RusT but that the sRNA also requires Hfq for the post-transcriptional regulation of target mRNAs.

**Fig 8:**
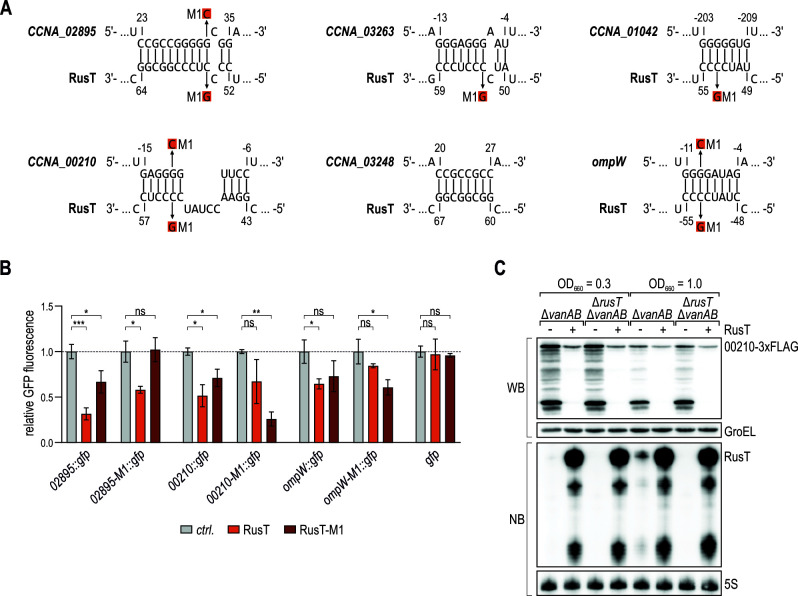
RusT employs a conserved sequence stretch for target recognition. (**A**) Base-pairing interactions between RusT and target mRNAs encoding transporters *CCNA_02895*, *CCNA_03263*, *CCNA_01042*, *CCNA_00210*, *CCNA_03248*, and *ompW* as predicted using the RNA hybrid and IntaRNA algorithms (56, 57). Nucleotide positions in the mRNA are marked relative to the start codon. The M1 (C53G) mutation in RusT and the corresponding M1 mutations in the mRNAs are indicated. (**B**) Regulation of target reporter fusions by RusT or RusT-M1. *C. crescentus* Δ*vanAB* Δ*rusT* cells carrying the indicated *gfp* reporter fusion in combination with either an empty control vector (pBV-MCS6; *ctrl*.), or the expression plasmid pP_van_-RusT or pP_van_-RusT-M1 were grown overnight in the presence of vanillate, and GFP expression was determined and quantified by fluorescence intensity measurements as described for Fig. 6. (**C**) CCNA_00210-3xFLAG protein expression in response to RusT overexpression. *C. crescentus* Δ*vanAB CCNA_00210::3xFLAG* cells carrying either an empty control vector [pBV-MCS6; (−)], or the expression plasmid pP_van_-RusT (+) were grown to mid-exponential and early stationary phase (OD_660_ of 0.3 and 1.0, respectively) in the presence of vanillate. Total protein samples were analyzed by Western blot to determine CCNA_00210–3xFLAG levels. Expression of RNA polymerase (RNAP) was probed as loading control. Induction of RusT from pP_van_-RusT was confirmed by Northern blot analysis; 5S rRNA served as loading control.

In analogy to regulatory proteins, sRNAs are assembled of modular domains that administrate either structural or functional roles (55). Typically, target recognition sites—referred to as seed sequences—are characterized by a high degree of sequence conservation between homologs, and low structural complexity. We thus assumed that an internal segment of RusT including the conserved sequence of the second stem-loop (nt 45–57; Fig. 2A) was relevant for base-pairing with target mRNAs. To test this hypothesis, we used the RNAhybrid and IntaRNA algorithms (56, 57) to predict interactions between the highly conserved RusT sequence element and the five TBDR-encoding and *ompW* mRNA portions present in the respective *gfp* reporter constructs (Fig. 8A). For five of the six target fusions, a conserved cytosine within the loop of the second hairpin of RusT (C53; Fig. 2B) was involved in the interaction. We thus inserted the relevant point mutation in pP_van_-RusT (C53G; RusT-M1) which did not affect the sRNA’s expression level (Fig. S9), and tested its effect on the regulation of the two TBDR reporters that were amenable to analysis via fluorescence intensity measurements, *02895::gfp* and *00210::gfp*, as well as on the *ompW::gfp* reporter. When compared to wild-type RusT, RusT-M1 displayed reduced GFP repression of the *02895::gfp* and *00210::gfp* fusions, indicating that the conserved C53 residue of the sRNA was involved in its regulatory activity in both cases (Fig. 8B). To restore target gene regulation, we next introduced compensatory single-point mutations in the respective reporter fusions (Fig. 8A). RusT had reduced capacity to repress *02895-M1::gfp*; however, we did not observe the restoration of the regulation in the presence of RusT-M1. In contrast, *00210-M1::gfp* and *ompW-M1::gfp* were specifically repressed by RusT-M1, and only partially amenable to regulation by the native RusT version (Fig. 8B).

As we had successfully confirmed that regulation of the *00210::gfp* target fusion was due to a direct, Hfq-dependent base-pairing interaction with RusT we tested whether the sRNA also affected the levels of the endogenous CCNA_00210 TBDR. We inserted a C-terminal 3xFLAG tag in the chromosomal copy of *CCNA_00210*, and compared protein levels in the presence and absence of RusT. *Caulobacter* wild-type and *rusT* mutant cells carrying either a control plasmid or the RusT overexpression construct pP_van_-RusT were grown in the presence of vanillate to mid-exponential and early stationary phase (OD_660_ of 0.3 and 1.0, respectively), and while the deletion of RusT did not significantly affect CCNA_00210 levels, we observed strongly reduced protein levels in the strain overexpressing RusT (Fig. 8C).

## DISCUSSION

### Specific binding of *Caulobacter* Hfq to RNA

Although Hfq was originally identified as a co-factor required for the replication of RNA phage Qβ in *E. coli* (58), the high degree of conservation in various bacterial species early on suggested an additional, more general physiological function of the RNA binding protein. Indeed, the primary role of Hfq is today seen in its complex contribution to post-transcriptional gene regulation, fostering the interaction between sRNAs and their cognate trans-encoded target mRNAs during stress responses (55). Base-pairing of sRNAs and mRNAs results in either positive or negative regulation of target translation and typically also affects transcript stability. The Hfq-governed networks are best studied in enterobacteria, where more than 100 sRNAs entertain thousands of individual interactions to modulate gene expression (10, 59). Hfq deficiency manifests in pleiotropic phenotypes including reduced growth, increased sensitivity to intrinsic and environmental stresses, alterations in central metabolism and reduced virulence of bacterial pathogens (60, 61). In *Caulobacter*, *hfq* null mutants suffer from severe defects in cell morphology and decreased tolerance to antibiotics targeting the cell wall (49), likely reflecting pervasive changes in gene expression and the accumulation of alpha-ketoglutarate which redirects peptidoglycan precursor metabolism. Hundreds of transcripts are deregulated in the absence of Hfq; however, the RNAs directly bound by Hfq are barely known (15, 49). In this study, we used co-immunoprecipitation of RNA with 3xFLAG-tagged Hfq protein in *C. crescentus* to capture the chaperone’s interactome in early stationary growth. In total, we recovered 311 transcripts including 21 non-coding RNAs with at least threefold enrichment in the co-IP sample when compared to the control (Fig. 1; Fig. S2; Table 1; Table S1). Of note, we also identified an increase in abundance of the two 23S rRNA transcripts (~17.5-fold and 22-fold for CCNA_R0084 and CCNA_R0066, respectively), while 16S and 5S rRNA transcripts were unaffected. As two distinct regions within the 23S transcripts were highly enriched, Hfq seems to interact specifically rather than spuriously with the transcripts. An association with stable house-keeping RNAs is not uncommon, *E. coli* Hfq interacts with 16S rRNA and r-protein S12 (62, 63), and is involved in the biogenesis of 30S ribosomes (64).

Binding of Hfq to client RNAs can be mediated via different domains. Six Hfq protomers assemble into a ring-like structure in which four different sites—the distal and proximal surfaces of the ring, the rim as well as the unstructured C-terminal domain—are available for interactions with RNA (65). Each binding site shows distinct preferences for RNA substrates and their differential usage enables Hfq to perform its regulatory functions. The distal surface typically interacts with purine-rich (ARN)_n_ motifs found in mRNA transcripts whereas sRNAs preferentially bind to the proximal face and the rim (66, 67). The flexible C-terminus is also able to contact the rim and has been implicated to restrict non-specific binding to Hfq (14). Initial recognition of sRNAs at the proximal face involves the 3′ uridine-rich stretch downstream of the Rho-independent terminator, a common feature of the 3′ ends of sRNAs (68, 69). In addition, Hfq frequently resides at AU-rich sequences just upstream the terminal hairpin (55). For RusT, our chemical probing experiments have confirmed an Hfq-specific foot-print at this position preceding the terminator (Fig. 2B and C), and deletion of the respective sequence patch in RusT-SL3mut greatly impeded Hfq-binding compared to the wild-type RNA (Fig. 1D and H). We likewise predicted the secondary structures of the 16 additional Hfq-binding sRNAs using RNAfold (70), and found a terminal hairpin structure for 12 transcripts, 10 of which were followed by a U-run (Table S3). When analyzed for the nucleotide composition of their sequences including 15 nt upstream of the sRNA terminators, we in almost all cases detected a lower GC content compared to the full molecule. Collectively, these results point toward a recognition mode by Hfq for at least a subset of sRNAs in *Caulobacter* that parallels the model established for enterobacterial species (55) However, alternative binding modes are possible, for example, when occupying sites at the distal face and the rim of Hfq.

### RusT is a trans-acting sRNA induced in response to iron starvation

Bacteria depend on iron as a critical and oftentimes scarce nutrient which is required as a cofactor in fundamental biological processes (28, 71). While iron-limited environments hence pose a serious constraint to survival, an excess of the metal can be toxic, and tight control of iron uptake, consumption and efflux to maintain metal homeostasis is necessary. In addition to transcription factors such as Fur, sRNAs have been repeatedly associated with the coordination of the cellular response to iron limitation (72).

**Fig 9:**
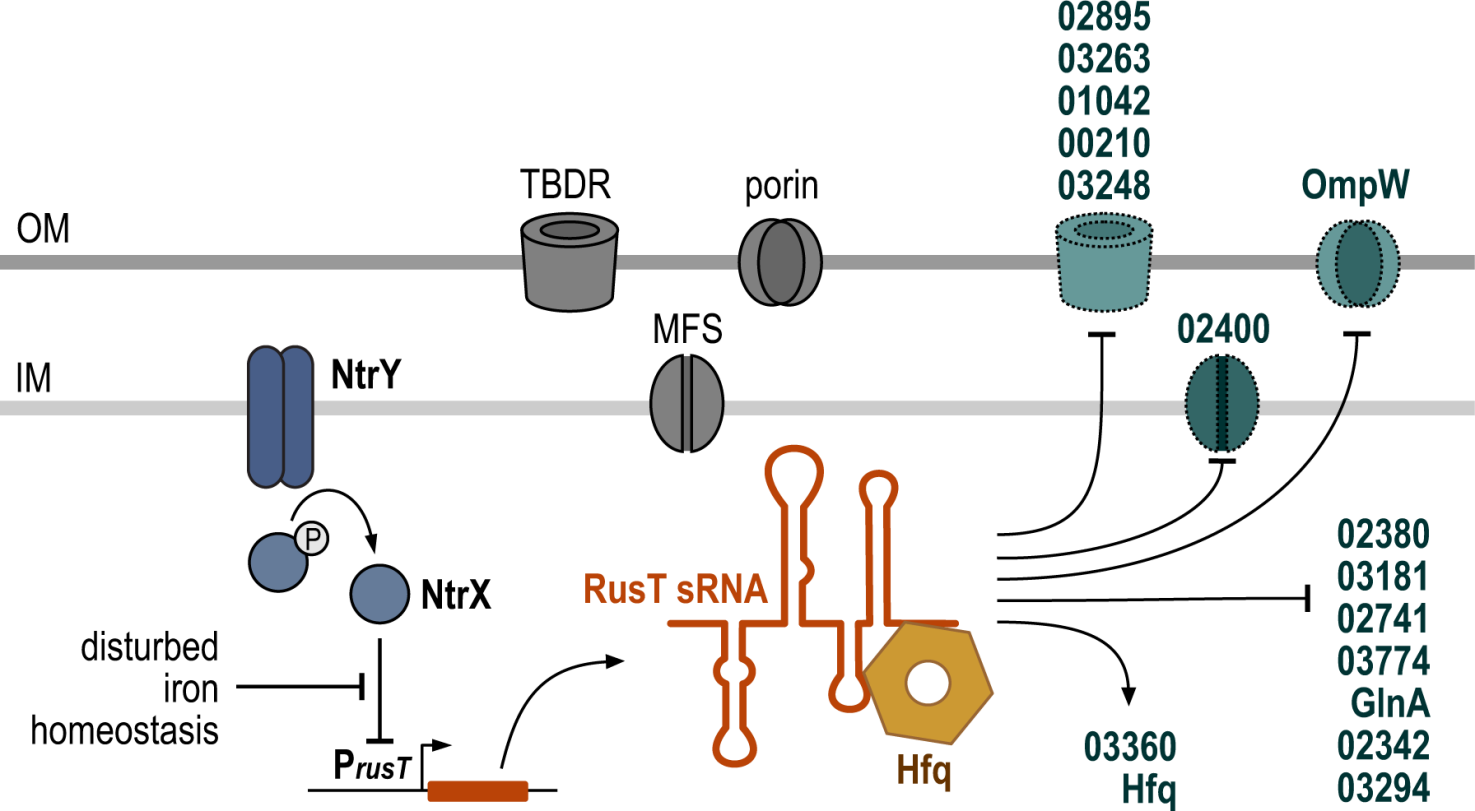
RusT expression and regulatory activity. Expression of RusT is under control of the NtrYX system and induced in response to disturbance of the iron homeostasis. Together with the RNA chaperone Hfq, RusT interacts with target mRNAs, including a number of transcripts encoding for different types of membrane-standing transporters.

The enterobacterial sRNA RyhB is only transcribed under iron-limited conditions and in conjunction with Hfq reduces the demand by redirecting iron to essential pathways and optimizing iron uptake. While RyhB and its functional analogues are Fur-dependent (73), it is intriguing that no sRNA has been identified in the Fur regulon in *Caulobacter. E. coli* RyhB functions as the non-coding switch to change the sign of regulation by Fur which acts as a repressor under iron-replete conditions (1). Different from *E. coli*, Fur has been demonstrated to also function as a direct transcriptional activator for a subset of genes in several organisms, including *N. meningitidis* (74) and *C. crescentus* (32). Its ability to act as a dual transcriptional regulator itself might alleviate the need for an sRNA within the direct Fur regulon. Other Fur-independent sRNAs, including FnrS and CyaR in the enterobacteria (72, 75), affect iron homeostasis and we identified the conserved Hfq-associated sRNA RusT (CCNA_R0199) to be synthesized under iron-limiting conditions and to function as an Hfq-dependent post-transcriptional regulator of 16 transcripts in *Caulobacter* (Fig. 6 and 9). When compared to the wild-type, a *rusT* deletion strain was not impaired for growth in the presence of DIP or ZnSO_4_ (Fig. S10), suggesting that the sRNA acts as a modulator rather than a central regulator of the stress response.

The largest functional class of RusT targets encode membrane proteins, such as the porin OmpW, the major facilitator family protein CCNA_02400 as well as the TBDRs CCNA_02895, CCNA_03263, CCNA_01042, CCNA_00210, and CCNA_03248 (Fig. 5C; Table 2). *Caulobacter* is equipped with an extraordinarily large set of outer membrane proteins, including more than 60 TBDRs which mediate gated, high-affinity uptake of vitamins, carbohydrates, and ferric siderophores (27, 76). The cell needs to constantly tune its envelope profile to optimize nutrient uptake in a given situation while at the same time avoid over-crowding and hence impairment of membrane integrity or fluidity. At the post-transcriptional level, sRNAs play a crucial role in regulation of outer membrane protein synthesis (1), and RusT joins the *Caulobacter* sRNAs CrfA and ChvR which both have been shown to affect TBDR expression in response to starvation and upon activation of the ChvGI TCS, respectively (17, 18). Except for *CCNA_02400* and *CCNA_03248*, expression of all the aforementioned transporter genes in the RusT regulon has also been identified to be downregulated in response to iron depletion in a previous transcriptome analysis (50), and RusT potentially contributes to this pattern through its regulatory activity at the post-transcriptional level. Similarly, also *CCNA_03181* (encoding an alcohol dehydrogenase) and *CCNA_03774* (encoding a citrate lyase beta chain/citryl-CoA lyase subunit) are repressed by RusT and in iron-scarce conditions [Fig. 6A and reference (50)].

### Transcriptional regulation of RusT

In this study, we demonstrated that RusT is induced in *Caulobacter* cells treated with the chelator DIP or by high amounts of zinc, two stressors that interfere with the cellular iron homeostasis (Fig. 3B). Transposon insertions in *ntrX*, encoding the response regulator of the conserved NtrYX TCS, abrogated stress regulation of RusT and resulted in constitutively high expression of the sRNA (Fig. 3C).

In *Caulobacter*, the *ntrYX* operon likely originated from gene duplication of the neighboring *ntrBC* locus; however, different from the latter one it has functionally not been linked to nitrogen metabolism (77). Unconventionally, NtrY is proposed to primarily act as a phosphatase on NtrX, and this activity is inhibited by a *Caulobacter*-specific periplasmic protein, NtrZ (47). Both functionally and genetically, NtrYX is linked to the ChvGI TCS. The two systems control partially overlapping networks to tune cell envelope integrity, and synergize to mediate growth under distinct environmental conditions (47, 78) involving dedicated sRNAs: ChvR is regulated by ChvI, while RusT is controlled by NtrX (Fig. 3C and D).

Homologs of the NtrYX TCS have been found in many alpha- and betaproteobacteria. While generally annotated to function in nitrogen assimilation, recent studies have reported the involvement of NtrYX in various additional cellular processes including the response to low oxygen tension in *Brucella abortus*, cell shape, motility, and biofilm formation in *Sinorhizobium meliloti* as well as cell envelope homeostasis in *Rhodobacter sphaeroides* (41–45). The NtrYX homolog of *P. denitrificans* responds to iron starvation, and inactivation of the TCS in this species results in the deregulation of factors involved in metal homeostasis and a subset of TBDRs (46). Our study likewise points toward a functional role of NtrYX in response to perturbations of the iron homeostasis, and a role for the sRNA RusT to aid membrane reorganization under this condition (Fig. 9).

RusT is also one of the *Caulobacter* sRNA candidates that shows differential regulation over the course of the cell cycle, with a peak of expression in swarmer and predivisional cells (21, 79). Involvement of several cell cycle regulators in the modulation of *rusT* promoter activity has either been predicted in bioinformatic analyses [two “half site motifs” for CtrA, two DnaA sites, one CcrM recognition motif (79)] or has been confirmed experimentally [ChIP-Seq peak for GcrA (21, 80); differential methylation in *mucR1/2* mutant cells (81)].

Iron deprivation has been identified to affect spatiotemporal localization of the division machinery and to interfere with peptidoglycan incorporation in dividing cells, thus blocking the late stages of cytokinesis (82). Hence, dissecting the transcriptional control of *rusT* might reveal a potential, RNA-mediated link between cell cycle control and iron homeostasis in *Caulobacter*.

## MATERIALS AND METHODS

### DNA oligonucleotides and plasmids

Sequences of all oligonucleotides employed in this study are listed in Table S4. All plasmids used in this study are summarized in Table S5, and details on plasmid construction are specified in the Supplemental Data file.

### Bacterial strains and growth conditions

Bacterial strains used in this study are listed in Table S6.

*C. crescentus* strain NA1000 (KFS-006) is referred to as the wild-type strain and was used for mutant generation. All details on strain construction and growth conditions are provided in the Supplemental Data file.

### Hfq co-IP and transcriptome analysis using RNA-seq

Details on the Hfq co-IP and the transcriptome analysis upon RusT overexpression using RNA-seq are provided in the Supplemental Data file.

### RNA isolation and Northern blot analysis

Total RNA from bacterial samples was prepared using the Hot Phenol method as described previously (17). For Northern blot analysis, 5–10 µg of total RNA were separated on 6–8% polyacrylamide (7 M urea) gels and electroblotted. Membranes were hybridized with gene-specific, 5′ end-labeled DNA-oligonucleotides at 42°C in Roti-Hybri-Quick hybridization solution (Roth), and washed in three subsequent steps with SSC wash buffers (5×/1×/0.5× SSC) supplemented with 0.1% SDS.

### *In vitro* RNA biochemistry

Details on the preparation of *in vitro* synthesized RNA and associated experiments are provided in the Supplemental Data file.

### Transposon screen

For genome-wide transposon mutagenesis of *C. crescentus* carrying a chromosomal P*_rusT_::nptII* fusion (integration of pKF776-1), the mini-*himar1* transposon conferring resistance to gentamycin (pMAR2xT7; (83)) was introduced via conjugation from the DAP-auxotroph *E. coli* WM3064 donor (84). Mutants with increased tolerance for kanamycin were selected on PYE plates supplemented with gentamycin (1 µg/mL) and kanamycin (20 µg/mL). Insertion sites were mapped by semi-random PCR as described previously (83) using transposon-specific primers KFO-2067 and KFO-2068 (nested), arbitrary primers KFO-0899 or KFO-0901 and KFO-0898 (nested) and sequencing primer KFO-2071.

### Chromatin immunoprecipitation coupled to deep sequencing (ChIP-Seq)

All details on ChIP-Seq with the *C. crescentus* CB15 *ntrX::ntrX-HA* strain are provided in the Supplemental Data file.

### Protein sample analysis

Whole-cell protein samples were prepared as described previously (17). Fusion proteins to GFP or the 3xFLAG epitope tag were detected using a monoclonal anti-FLAG antibody (1:1,000; mouse; Sigma #F1804) or an anti-GFP antibody (1:1,000; mouse, Roche #11814460001), respectively. RNA polymerase (1:10,000; rabbit; BioLegend #663205) or GroEL (1:20,000; rabbit; Merck; #G6532) served as a loading controls.

### Fluorescence intensity measurements

GFP expression of translational reporter fusions was determined from cells cultivated overnight in PYE supplemented with the appropriate antibiotics and supplements. Upon collection of samples by centrifugation (3 min; 9,000 rpm; 4°C), the cells were washed once in 1× phosphate buffer, and resuspended in phosphate buffer. Background fluorescence was determined by a control sample not expressing GFP. Fluorescence intensity in the presence of the sRNA control plasmid was set to 1, and relative expression was calculated from three biological replicates (error bars represent standard deviation). Statistical significance was determined using unpaired *t* tests with Welch correction. The *P* value is indicated as follows—ns for *P* > 0.05, * for *P* ≤ 0.05, ** for *P* ≤ 0.01, *** for *P* ≤ 0.001, and **** for *P* ≤ 0.0001.

## Data Availability

Sequencing data have been deposited at the National Center for Biotechnology Information’s Gene Expression Omnibus (GEO) repository (85) and are available via the GEO accession numbers GSE148206 (Hfq co-IP), GSE148208 (RusT pulse overexpression), and GSE247928 (ChIP-seq).

## SUPPLEMENTAL MATERIAL

Table S1 mbio.03153-23-s0001.docx Hfq-associated transcripts.

Table S2 mbio.03153-23-s0002.xlsx NtrX-HA ChIP-seq analysis.

Supporting Information mbio.03153-23-s0003.pdf Figures S1 to S10, Tables S3 to S6, and supporting methodology.

ASM does not own the copyrights to Supplemental Material that may be linked to, or accessed through, an article. The authors have granted ASM a non-exclusive, world-wide license to publish the Supplemental Material files. Please contact the corresponding author directly for reuse.
