# Supplementary material for: Genome-wide profiling of Hfq-bound RNAs reveals the iron-responsive small RNA RusT in Caulobacter crescentus
Source: mBio. 2024 Mar 21;15(4):e03153-23. doi: 10.1128/mbio.03153-23 (PMC11005374; doi:10.1128/mbio.03153-23)
Supplement: Table S1 — Hfq-associated transcripts. [file mbio.03153-23-s0001.docx]

**Supplementary Table S1 – Hfq-associated transcripts (≥ 3-fold enrichment)**

| gene | enrichment | FDR p-value | annotation |
| --- | --- | --- | --- |
| CCNA_R0093 | 172,1 | 9,5E-80 | small non-coding RNA crfA |
| CCNA_00834 | 83,6 | 6,1E-142 | flagellin FljM |
| CCNA_R0199 | 73,8 | 5,8E-150 | small non-coding RNA |
| CCNA_R0157 | 63,8 | 1,8E-111 | small non-coding RNA |
| CCNA_R0014 | 51,7 | 3,4E-53 | stationary phase expressed sRNA |
| CCNA_00835 | 51,0 | 4,0E-117 | flagellin FljN |
| CCNA_R0188 | 49,2 | 1,4E-120 | small non-coding RNA |
| CCNA_01819 | 43,6 | 6,7E-98 | RNA-binding protein, Hfq family |
| CCNA_03400 | 40,1 | 1,8E-112 | conserved hypothetical protein |
| CCNA_01101 | 36,7 | 2,2E-110 | translation initiation factor 3 (IF-3) |
| CCNA_R0143 | 34,9 | 8,2E-32 | small non-coding RNA |
| CCNA_03360 | 34,4 | 1,7E-120 | tagatose-1,6-bisphosphate aldolase |
| CCNA_03467 | 33,8 | 7,7E-113 | zinc-dependent metalloprotease, MMP subfamily 2 |
| CCNA_01120 | 29,7 | 2,9E-39 | hypothetical protein |
| CCNA_02484 | 26,3 | 3,8E-74 | trans-feruloyl-CoA hydratase |
| CCNA_03313 | 22,5 | 1,1E-45 | hypothetical protein |
| CCNA_R0066 | 22,0 | 4,0E-88 | 23S ribosomal RNA |
| CCNA_02534 | 21,2 | 2,6E-89 | hypothetical protein |
| CCNA_R0097 | 20,7 | 1,9E-63 | small non-coding RNA |
| CCNA_02036 | 20,6 | 2,1E-87 | DNA-binding protein HU |
| CCNA_01043 | 19,6 | 3,6E-75 | hypothetical protein |
| CCNA_00691 | 19,5 | 2,1E-43 | ferredoxin |
| CCNA_03984 | 18,9 | 1,5E-65 | hypothetical protein |
| CCNA_02543 | 18,7 | 5,2E-84 | LSU ribosomal protein L33P |
| CCNA_03348 | 18,5 | 2,3E-75 | conserved hypothetical protein |
| CCNA_00987 | 18,2 | 1,6E-76 | stress response protein CsbD |
| CCNA_03959 | 18,1 | 7,4E-68 | hypothetical protein |
| CCNA_R0195 | 17,6 | 1,7E-63 | small non-coding RNA |
| CCNA_R0084 | 17,5 | 2,7E-77 | 23S ribosomal RNA |
| CCNA_01505 | 16,1 | 1,2E-65 | hypothetical protein |
| CCNA_02780 | 15,8 | 2,9E-74 | hypothetical protein |
| CCNA_02223 | 15,3 | 8,3E-66 | beta-lactamase, type II |
| CCNA_01044 | 15,3 | 2,4E-50 | AcrR-family transcriptional regulator |
| CCNA_00994 | 15,1 | 2,7E-38 | oxidoreductase, GMC family |
| CCNA_00836 | 15,0 | 5,4E-79 | flagellin FljO |
| CCNA_02963 | 14,9 | 1,6E-60 | conserved hypothetical protein |
| CCNA_03405 | 14,4 | 1,1E-54 | rpsU-divergently transcribed protein |
| CCNA_01592 | 13,8 | 1,1E-67 | SN-glycerol-3-phosphate transport ATP-binding protein |
| CCNA_02174 | 13,6 | 3,7E-79 | multidrug resistance efflux pump |
| CCNA_02838 | 13,6 | 1,1E-74 | EF hand domain protein |
| CCNA_01290 | 13,5 | 4,2E-47 | hypothetical protein |
| CCNA_01610 | 13,4 | 5,3E-69 | 2-isopropylmalate synthase |
| CCNA_02616 | 12,0 | 3,5E-84 | 4-hydroxyphenylpyruvate dioxygenase |
| CCNA_02328 | 11,7 | 1,8E-69 | cell cycle regulatory protein GcrA |
| CCNA_02483 | 11,7 | 4,8E-66 | 4-coumarate--CoA ligase |
| CCNA_03267 | 11,5 | 1,1E-71 | HipB-related transcriptional regulatory protein |
| CCNA_03263 | 11,4 | 7,7E-75 | TonB-dependent receptor |
| CCNA_03639 | 11,4 | 2,5E-66 | ferredoxin, 2Fe-2s |
| CCNA_R0040 | 11,4 | 1,9E-40 | small non-coding RNA |
| CCNA_03374 | 11,3 | 4,4E-69 | hypothetical protein |
| CCNA_02709 | 10,9 | 3,8E-64 | CBS domain containing protein |
| CCNA_01361 | 10,9 | 1,6E-67 | PepSY superfamily protein |
| CCNA_02576 | 10,5 | 3,3E-67 | phosphoribosylamidoimidazole-succinocarboxamide synthase |
| CCNA_02716 | 9,9 | 2,0E-61 | ABC transporter substrate-binding protein |
| CCNA_00034 | 9,9 | 1,4E-40 | SSU ribosomal protein S15P |
| CCNA_00592 | 9,8 | 6,3E-69 | ferritin superfamily protein |
| CCNA_01418 | 9,5 | 9,4E-63 | ROS/MUCR transcriptional regulator |
| CCNA_01725 | 9,4 | 4,9E-59 | beta-barrel assembly machine (BAM) protein BamB |
| CCNA_03593 | 9,3 | 8,1E-71 | integral membrane protein |
| CCNA_02309 | 9,1 | 1,7E-45 | EF hand domain protein |
| CCNA_R0133 | 9,1 | 1,0E-41 | small non-coding RNA |
| CCNA_03060 | 9,0 | 1,9E-56 | conserved hypothetical protein |
| CCNA_00543 | 9,0 | 7,6E-44 | methyl-accepting chemotaxis protein |
| CCNA_01852 | 8,7 | 1,9E-30 | major facilitator superfamily transporter |
| CCNA_02817 | 8,7 | 9,3E-52 | hypothetical protein |
| CCNA_02542 | 8,7 | 3,7E-38 | hypothetical protein |
| CCNA_02763 | 8,6 | 1,6E-61 | bacteriophage P4 integrase |
| CCNA_03403 | 8,5 | 1,1E-45 | small coiled-coil domain protein |
| CCNA_01705 | 8,4 | 3,0E-59 | conserved hypothetical protein |
| CCNA_03359 | 8,3 | 8,0E-60 | phosphoglycerate kinase |
| CCNA_02747 | 8,2 | 3,2E-51 | ABC transporter substrate-binding protein |
| CCNA_01017 | 8,2 | 5,2E-60 | acyl-CoA synthetase |
| CCNA_R0025 | 8,1 | 1,2E-29 | small non-coding RNA |
| CCNA_02141 | 8,1 | 3,3E-59 | flagellar fliL protein |
| CCNA_03629 | 8,0 | 7,5E-60 | DNA replication inhibitor toxin SocB |
| CCNA_00808 | 7,9 | 8,5E-24 | LSU ribosomal protein L34P |
| CCNA_03695 | 7,8 | 1,5E-25 | aldehyde dehydrogenase |
| CCNA_03459 | 7,7 | 1,6E-20 | methyl-accepting chemotaxis protein |
| CCNA_00530 | 7,7 | 2,8E-23 | LSU ribosomal protein L10P |
| CCNA_01319 | 7,6 | 1,4E-23 | SSU ribosomal protein S14P, RpsN |
| CCNA_00245 | 7,5 | 1,5E-26 | pterin-4-alpha-carbinolamine dehydratase |
| CCNA_01696 | 7,4 | 3,3E-33 | aminobenzoyl-glutamate transport protein |
| CCNA_03529 | 7,4 | 9,2E-42 | polyphosphate kinase 2 |
| CCNA_01296 | 7,4 | 1,2E-62 | stalk-specific protein X |
| CCNA_01652 | 7,4 | 3,2E-56 | phosphopantetheine adenylyltransferase |
| CCNA_03234 | 7,3 | 4,4E-54 | glutamine amidotransferase, class I |
| CCNA_03622 | 7,3 | 2,2E-22 | beta-lactamase repressor |
| CCNA_03859 | 7,2 | 5,2E-61 | two-component response regulator cenR |
| CCNA_00919 | 7,2 | 5,2E-44 | hypothetical protein |
| CCNA_01287 | 7,2 | 3,3E-54 | epoxide hydrolase |
| CCNA_01317 | 7,2 | 1,7E-27 | LSU ribosomal protein L24P, RplX |
| CCNA_03767 | 7,2 | 1,2E-41 | SSU ribosomal protein S16P |
| CCNA_01759 | 7,1 | 2,8E-44 | peptidyl-prolyl cis-trans isomerase |
| CCNA_00092 | 7,1 | 3,9E-41 | short chain dehydrogenase |
| CCNA_R0127 | 7,0 | 5,4E-44 | small non-coding RNA |
| CCNA_02816 | 7,0 | 3,5E-44 | hypothetical protein |
| CCNA_01625 | 6,9 | 1,2E-27 | aminobenzoyl-glutamate utilization protein B |
| CCNA_01977 | 6,9 | 8,3E-19 | 1-acyl-sn-glycerol-3-phosphate acyltransferase |
| CCNA_01475 | 6,8 | 2,8E-24 | OmpW family outer membrane protein |
| CCNA_02461 | 6,8 | 1,2E-17 | conserved hypothetical protein |
| CCNA_03947 | 6,8 | 4,7E-16 | hypothetical protein |
| CCNA_01330 | 6,7 | 1,2E-27 | DNA-directed RNA polymerase alpha chain, RpoA |
| CCNA_01236 | 6,7 | 3,8E-17 | general stress protein GsiB |
| CCNA_00573 | 6,7 | 5,5E-27 | TonB-dependent receptor |
| CCNA_00946 | 6,6 | 4,1E-40 | Basal-body rod modification protein FlgD |
| CCNA_02027 | 6,6 | 4,8E-44 | NADH-quinone oxidoreductase chain E, NuoE |
| CCNA_03414 | 6,5 | 4,0E-60 | Rubrum transdehydrogenase NAD-binding |
| CCNA_01866 | 6,5 | 5,2E-32 | cation/multidrug efflux pump acrB4 |
| CCNA_00701 | 6,5 | 2,1E-41 | cold shock protein CspB |
| CCNA_03379 | 6,4 | 1,1E-22 | Cro/CI family transcriptional regulator |
| CCNA_02895 | 6,4 | 1,0E-39 | TonB-dependent receptor |
| CCNA_01820 | 6,4 | 3,2E-41 | GTP-binding protein hflX |
| CCNA_00153 | 6,4 | 9,2E-41 | adenine nucleotide exchange factor GrpE |
| CCNA_02748 | 6,3 | 1,4E-23 | conserved hypothetical protein |
| CCNA_03879 | 6,2 | 5,0E-32 | uroporphyrinogen decarboxylase, HemE |
| CCNA_00568 | 6,2 | 5,1E-27 | N-acetylglucosamine-6-phosphate deacetylase |
| CCNA_00531 | 6,2 | 1,0E-18 | LSU ribosomal protein L12P (L7/L12) |
| CCNA_00162 | 6,1 | 8,9E-33 | conserved hypothetical protein |
| CCNA_01084 | 6,0 | 1,3E-27 | hypothetical protein |
| CCNA_01047 | 6,0 | 4,3E-35 | TonB-dependent receptor |
| CCNA_02652 | 6,0 | 1,1E-38 | cyclohexanone monooxygenase |
| CCNA_00982 | 6,0 | 3,5E-30 | ROS/MUCR transcriptional regulator |
| CCNA_00375 | 6,0 | 1,2E-35 | conserved hypothetical protein |
| CCNA_01142 | 5,9 | 2,0E-21 | hypothetical protein |
| CCNA_03231 | 5,9 | 6,9E-41 | anti-toxin protein relB-4 |
| CCNA_01528 | 5,8 | 8,2E-32 | flagellin fljK |
| CCNA_02725 | 5,8 | 8,7E-23 | choline dehydrogenase |
| CCNA_01410 | 5,8 | 6,7E-23 | cyclohexanone monooxygenase |
| CCNA_R0098 | 5,7 | 1,0E-18 | small non-coding RNA |
| CCNA_01424 | 5,7 | 1,6E-19 | hypothetical protein |
| CCNA_03791 | 5,7 | 1,1E-18 | MarR-family transcriptional regulator |
| CCNA_01019 | 5,7 | 1,5E-27 | beta-glucosidase |
| CCNA_R0156 | 5,6 | 3,1E-14 | small non-coding RNA |
| CCNA_02235 | 5,6 | 7,7E-17 | SGNH hydrolase family protein |
| CCNA_01621 | 5,6 | 1,4E-25 | DNA-directed RNA polymerase omega chain |
| CCNA_02607 | 5,6 | 5,2E-14 | isocitrate dehydrogenase |
| CCNA_01944 | 5,6 | 4,3E-27 | McrA-family restriction endonuclease |
| CCNA_02191 | 5,6 | 2,5E-27 | hypothetical protein |
| CCNA_02843 | 5,6 | 5,0E-32 | toxin protein parE-3 |
| CCNA_03573 | 5,6 | 1,9E-24 | D-aminoacylase |
| CCNA_03385 | 5,5 | 4,0E-27 | flagellin N-methylase |
| CCNA_01583 | 5,5 | 4,6E-25 | phosphate-binding protein |
| CCNA_00465 | 5,4 | 1,0E-28 | UDP-galactopyranose mutase |
| CCNA_03574 | 5,4 | 9,9E-21 | TonB-dependent receptor |
| CCNA_00496 | 5,4 | 1,1E-27 | threonyl-tRNA synthetase |
| CCNA_03105 | 5,3 | 2,8E-25 | DnaJ domain protein |
| CCNA_02410 | 5,3 | 1,3E-14 | alpha-methylacyl-CoA racemase |
| CCNA_00341 | 5,3 | 1,2E-26 | succinyl-CoA synthetase alpha chain, SucD |
| CCNA_02207 | 5,2 | 1,1E-27 | conserved hypothetical protein |
| CCNA_03587 | 5,2 | 2,1E-13 | entericidin B-like protein |
| CCNA_R0049 | 5,1 | 7,3E-10 | tmRNA |
| CCNA_R0016 | 5,0 | 1,3E-15 | small non-coding RNA |
| CCNA_03476 | 5,0 | 7,1E-09 | ribonuclease HI |
| CCNA_00396 | 4,9 | 5,7E-16 | conserved hypothetical protein |
| CCNA_00007 | 4,9 | 7,1E-22 | SSU ribosomal protein S20P |
| CCNA_02111 | 4,9 | 1,9E-20 | pilF-related tetratricopeptide repeat protein |
| CCNA_01004 | 4,9 | 8,4E-28 | flagellar basal-body rod protein FlgB |
| CCNA_01521 | 4,9 | 2,9E-24 | putative exported protein |
| CCNA_00466 | 4,8 | 3,6E-26 | glycosyltransferase |
| CCNA_01267 | 4,8 | 5,5E-24 | NTF2 superfamily protein |
| CCNA_03232 | 4,8 | 6,5E-27 | toxin protein relE-4 |
| CCNA_01318 | 4,8 | 6,8E-14 | LSU ribosomal protein L5P, RplE |
| CCNA_00761 | 4,8 | 8,0E-23 | conserved hypothetical protein |
| CCNA_00537 | 4,8 | 7,8E-20 | DNA-directed RNA polymerase beta' chain |
| CCNA_03169 | 4,8 | 2,9E-22 | SH3 domain protein |
| CCNA_03239 | 4,8 | 2,4E-21 | spermidine/putrescine-binding protein |
| CCNA_02913 | 4,8 | 5,9E-24 | conserved hypothetical protein |
| CCNA_01083 | 4,7 | 1,4E-25 | HATPase superfamily protein |
| CCNA_01561 | 4,7 | 1,1E-09 | hypothetical protein |
| CCNA_01462 | 4,7 | 1,5E-25 | lactate 2-monooxygenase |
| CCNA_01909 | 4,7 | 2,5E-21 | ABC transporter substrate-binding protein |
| CCNA_01393 | 4,7 | 3,9E-21 | soluble lytic murein transglycosylase |
| CCNA_03061 | 4,6 | 1,5E-23 | SDR family dehydrogenase, group 9 |
| CCNA_02232 | 4,5 | 2,2E-08 | TonB-dependent receptor |
| CCNA_02480 | 4,5 | 6,6E-22 | NAD-dependent benzaldehyde dehydrogenase II |
| CCNA_02555 | 4,5 | 1,6E-14 | adenosine 5'-monophosphoramidase |
| CCNA_03576 | 4,5 | 3,0E-13 | hypothetical protein |
| CCNA_02011 | 4,5 | 2,2E-20 | methylmalonyl CoA epimerase |
| CCNA_02085 | 4,5 | 1,8E-19 | anhydromuramoyl-peptide exo-beta-N-acetylglucosaminidase |
| CCNA_03949 | 4,5 | 1,2E-25 | conserved hypothetical protein |
| CCNA_01967 | 4,5 | 1,4E-20 | hypothetical protein with pentapeptide repeats |
| CCNA_03334 | 4,5 | 7,2E-20 | cell division protein FtsH |
| CCNA_00079 | 4,5 | 7,2E-26 | MerR-family transcriptional regulator |
| CCNA_03978 | 4,5 | 1,9E-11 | hypothetical protein |
| CCNA_03332 | 4,4 | 1,0E-14 | dihydropteroate synthase |
| CCNA_01498 | 4,4 | 1,3E-24 | DNA-dependent DNA polymerase III alpha chain |
| CCNA_00328 | 4,4 | 6,4E-24 | monofunctional biosynthetic peptidoglycan transglycosylase MtgA |
| CCNA_01841 | 4,4 | 3,4E-24 | isocitrate lyase |
| CCNA_00636 | 4,4 | 2,7E-20 | hypothetical protein |
| CCNA_02178 | 4,3 | 1,5E-22 | short-chain alcohol dehydrogenase |
| CCNA_00451 | 4,3 | 8,9E-22 | TonB-dependent receptor |
| CCNA_R0184 | 4,3 | 2,3E-16 | small non-coding RNA |
| CCNA_02465 | 4,3 | 3,1E-15 | UDP-glucose 6-dehydrogenase |
| CCNA_02564 | 4,2 | 9,4E-26 | acyl-CoA dehydrogenase, short-chain specific |
| CCNA_02090 | 4,2 | 1,2E-25 | exodeoxyribonuclease III |
| CCNA_00243 | 4,2 | 4,3E-10 | hypothetical protein |
| CCNA_00607 | 4,2 | 3,2E-15 | TonB-dependent receptor |
| CCNA_03099 | 4,2 | 1,5E-12 | hypothetical protein |
| CCNA_00817 | 4,2 | 4,9E-23 | L-sorbosone dehydrogenase |
| CCNA_03001 | 4,2 | 2,4E-17 | conserved hypothetical protein |
| CCNA_03181 | 4,2 | 3,5E-22 | alcohol dehydrogenase |
| CCNA_00199 | 4,2 | 4,7E-22 | TadG/vWA-family protein |
| CCNA_00338 | 4,1 | 2,7E-21 | TonB-dependent receptor |
| CCNA_02997 | 4,1 | 1,4E-15 | cold shock protein CspA |
| CCNA_02155 | 4,1 | 1,2E-15 | conserved hypothetical protein |
| CCNA_01383 | 4,1 | 1,9E-09 | conserved hypothetical protein |
| CCNA_03582 | 4,1 | 1,6E-15 | 3'(2'),5'-bisphosphate nucleotidase CysQ |
| CCNA_02773 | 4,1 | 5,2E-09 | methyl-accepting chemotaxis protein |
| CCNA_03904 | 4,1 | 1,1E-18 | hypothetical protein |
| CCNA_02059 | 4,1 | 4,7E-19 | ribosomal large subunit pseudouridine synthase C |
| CCNA_01021 | 4,0 | 8,6E-22 | TonB-dependent receptor |
| CCNA_03444 | 4,0 | 2,7E-17 | TonB-dependent receptor |
| CCNA_03446 | 4,0 | 2,4E-16 | feruloyl-CoA synthetase |
| CCNA_02160 | 4,0 | 4,6E-11 | hypothetical protein |
| CCNA_01376 | 4,0 | 4,9E-22 | glutathione S-transferase |
| CCNA_01423 | 4,0 | 2,9E-19 | thiamine-monophosphate kinase |
| CCNA_01311 | 4,0 | 5,7E-11 | LSU ribosomal protein L22P, RplV |
| CCNA_00657 | 3,9 | 1,6E-21 | type I restriction-modification system specificity subunit |
| CCNA_03662 | 3,9 | 2,9E-11 | hypothetical protein |
| CCNA_02082 | 3,9 | 4,2E-20 | Sec-independent protein translocase protein tatA |
| CCNA_03293 | 3,9 | 7,0E-22 | multifunctional fatty acid oxidation complex subunit alpha FadJ |
| CCNA_00676 | 3,9 | 6,0E-13 | PSK-family transcription factor |
| CCNA_02185 | 3,9 | 8,6E-12 | acetolactate synthase large subunit |
| CCNA_02783 | 3,9 | 3,6E-18 | two-component response regulator |
| CCNA_01316 | 3,9 | 2,4E-18 | LSU ribosomal protein L14P, RplN |
| CCNA_02037 | 3,9 | 1,6E-19 | ATP-dependent endopeptidase Lon |
| CCNA_02677 | 3,8 | 2,8E-06 | glutathione S-transferase |
| CCNA_03577 | 3,8 | 4,5E-19 | DNA polymerase I |
| CCNA_01315 | 3,8 | 2,6E-15 | SSU ribosomal protein S17P, RpsQ |
| CCNA_01785 | 3,8 | 9,7E-08 | hypothetical protein |
| CCNA_02341 | 3,8 | 1,3E-18 | small heat shock protein |
| CCNA_00403 | 3,8 | 4,2E-21 | short chain dehydrogenase |
| CCNA_01741 | 3,8 | 4,8E-10 | SSU ribosomal protein S6P |
| CCNA_01085 | 3,8 | 1,8E-21 | DNA-cytosine methyltransferase |
| CCNA_01263 | 3,7 | 3,2E-18 | pyruvate dehydrogenase E1 component |
| CCNA_01802 | 3,7 | 7,5E-08 | hypothetical protein |
| CCNA_03632 | 3,7 | 4,5E-13 | hypothetical protein |
| CCNA_02400 | 3,7 | 2,1E-13 | transporter, major facilitator superfamily |
| CCNA_01386 | 3,6 | 5,8E-18 | conserved hypothetical protein |
| CCNA_01522 | 3,6 | 4,7E-16 | ATP-dependent protease subunit |
| CCNA_02663 | 3,6 | 1,6E-17 | DnaK suppressor protein DksA |
| CCNA_01527 | 3,6 | 3,7E-14 | flagellin fljL |
| CCNA_02460 | 3,5 | 1,0E-19 | conserved hypothetical protein |
| CCNA_03766 | 3,5 | 1,3E-10 | 16S rRNA processing protein RimM |
| CCNA_00048 | 3,5 | 8,6E-18 | S-adenosylmethionine synthetase, MetK |
| CCNA_00735 | 3,5 | 9,1E-16 | beta-barrel assembly machine (BAM) protein BamF |
| CCNA_03228 | 3,5 | 8,9E-18 | tetratricopeptide repeat family protein |
| CCNA_02923 | 3,5 | 8,9E-10 | TonB-dependent receptor |
| CCNA_00522 | 3,5 | 4,5E-16 | BrnT-related ribonuclease toxin |
| CCNA_03447 | 3,4 | 2,2E-17 | hypothetical protein |
| CCNA_02774 | 3,4 | 1,7E-19 | hypothetical protein |
| CCNA_02298 | 3,4 | 2,7E-19 | MJ0042 family finger-like domain protein |
| CCNA_01421 | 3,4 | 4,6E-16 | 6,7-dimethyl-8-ribityllumazine synthase |
| CCNA_R0175 | 3,4 | 3,6E-15 | small non-coding RNA |
| CCNA_00053 | 3,4 | 1,6E-13 | phosphate starvation-inducible protein PhoH |
| CCNA_01139 | 3,4 | 2,2E-14 | conserved hypothetical cytosolic protein |
| CCNA_01901 | 3,4 | 3,5E-16 | lipoprotein |
| CCNA_00041 | 3,4 | 9,7E-14 | protein translation initiation factor 2 IF-2 |
| CCNA_01382 | 3,4 | 2,7E-16 | long-chain-fatty-acid--CoA ligase |
| CCNA_02832 | 3,4 | 5,4E-19 | microsomal dipeptidase |
| CCNA_01329 | 3,4 | 3,8E-16 | SSU ribosomal protein S11P, RpsK |
| CCNA_01638 | 3,4 | 9,3E-14 | beta-lactamase family protein |
| CCNA_02091 | 3,4 | 1,9E-16 | conserved hypothetical protein |
| CCNA_01530 | 3,4 | 6,8E-05 | flagellin FljJ |
| CCNA_03557 | 3,4 | 2,2E-13 | conserved hypothetical protein |
| CCNA_00656 | 3,4 | 7,3E-16 | type I restriction-modification system methylation subunit |
| CCNA_02262 | 3,3 | 4,5E-08 | laminin G domain protein |
| CCNA_00958 | 3,3 | 1,0E-04 | conserved hypothetical protein |
| CCNA_03775 | 3,3 | 1,3E-12 | conserved hypothetical protein |
| CCNA_R0117 | 3,3 | 4,5E-11 | small non-coding RNA |
| CCNA_02176 | 3,3 | 9,0E-09 | hypothetical protein |
| CCNA_00811 | 3,3 | 6,0E-09 | TetR-family transcriptional regulator |
| CCNA_00469 | 3,3 | 6,1E-16 | GT1-family glycosyltransferase |
| CCNA_00342 | 3,3 | 1,3E-12 | 2-oxoglutarate dehydrogenase E1 component, OdhA |
| CCNA_03324 | 3,3 | 1,8E-14 | hypothetical protein |
| CCNA_01797 | 3,3 | 6,8E-17 | FtsB-related septum formation initiator |
| CCNA_03630 | 3,3 | 5,8E-18 | antitoxin protein SocA |
| CCNA_00598 | 3,3 | 4,4E-08 | TonB-dependent receptor |
| CCNA_00157 | 3,3 | 1,3E-08 | glyoxalase family protein |
| CCNA_00986 | 3,2 | 9,2E-14 | general stress protein 17O |
| CCNA_01425 | 3,2 | 3,8E-12 | H+ translocating pyrophosphatase |
| CCNA_02547 | 3,2 | 2,1E-12 | response regulator receiver protein DivK |
| CCNA_03768 | 3,2 | 6,9E-09 | signal recognition particle GTPase, SRP |
| CCNA_02741 | 3,2 | 1,4E-12 | conserved hypothetical protein |
| CCNA_01030 | 3,2 | 1,5E-06 | peptidyl-prolyl cis-trans isomerase |
| CCNA_00045 | 3,2 | 7,9E-16 | inorganic pyrophosphatase |
| CCNA_01830 | 3,2 | 8,6E-15 | TonB-dependent receptor |
| CCNA_01077 | 3,2 | 2,4E-13 | penicillin acylase |
| CCNA_00064 | 3,2 | 4,2E-09 | methyl-accepting chemotaxis protein |
| CCNA_01322 | 3,1 | 2,0E-10 | LSU ribosomal protein L18P, RplR |
| CCNA_02467 | 3,1 | 2,5E-15 | polyisoprenylphosphate hexose-1-phosphotransferase pssZ |
| CCNA_02495 | 3,1 | 1,8E-07 | 4-hydroxybenzoate transporter |
| CCNA_03311 | 3,1 | 9,0E-11 | protein translocase subunit SecE |
| CCNA_03305 | 3,1 | 1,9E-15 | SSU ribosomal protein S7P |
| CCNA_03702 | 3,1 | 3,3E-13 | SSU ribosomal protein S1P |
| CCNA_03820 | 3,1 | 2,8E-13 | outer-membrane lipoproteins carrier protein |
| CCNA_03931 | 3,1 | 1,2E-13 | hypothetical protein |
| CCNA_00776 | 3,1 | 1,5E-06 | OAR protein precursor |
| CCNA_03955 | 3,1 | 7,7E-05 | hypothetical protein |
| CCNA_02899 | 3,1 | 5,7E-13 | tryptophan halogenase |
| CCNA_01684 | 3,1 | 9,6E-13 | phenylalanine-4-hydroxylase |
| CCNA_00875 | 3,1 | 1,5E-04 | Flp/Fap pilin component protein |
| CCNA_02681 | 3,1 | 6,7E-12 | tRNA (uracil-5-)-methyltransferase GidA |
| CCNA_03641 | 3,1 | 1,1E-12 | succinate dehydrogenase iron-sulfur protein |
| CCNA_03397 | 3,1 | 1,6E-07 | conserved coiled-coil domain protein |
| CCNA_02476 | 3,0 | 8,7E-08 | vanillate demethylase oxygenase subunit A vanA |
| CCNA_02931 | 3,0 | 3,7E-06 | flgE-related flagellar hook protein |
| CCNA_01886 | 3,0 | 3,3E-07 | TonB-dependent receptor |
| CCNA_03266 | 3,0 | 3,5E-10 | conserved hypothetical protein |
| CCNA_00659 | 3,0 | 3,9E-12 | type I restriction-modification system restriction subunit |
| CCNA_00545 | 3,0 | 1,8E-11 | acetoacetyl-CoA reductase |
