# Supplementary material for: Genome-wide profiling of Hfq-bound RNAs reveals the iron-responsive small RNA RusT in Caulobacter crescentus
Source: mBio. 2024 Mar 21;15(4):e03153-23. doi: 10.1128/mbio.03153-23 (PMC11005374; doi:10.1128/mbio.03153-23)
Supplement: Supporting Information — Figures S1 to S10, Tables S3 to S6, and supporting methodology. [file mbio.03153-23-s0003.pdf]

Supporting Information for

Genome-wide profiling of Hfq-bound RNAs reveals the iron-responsive small RNA RusT in *Caulobacter crescentus*

Laura N. Vogt, Gaël Panis, Anna Schäpers, Nikolai Peschek, Michaela Huber, Kai Papenfort, Patrick H. Viollier, Kathrin S. Fröhlich

corresponding author: Kathrin Sophie Fröhlich
Email: kathrin.froehlich@uni-jena.de

This PDF file includes:

Figures S1 to S10
Tables S3-S6
Supporting Methodology
SI References

Other supporting materials for this manuscript include the following:

Tables S1, S2

Supplementary Figure S1

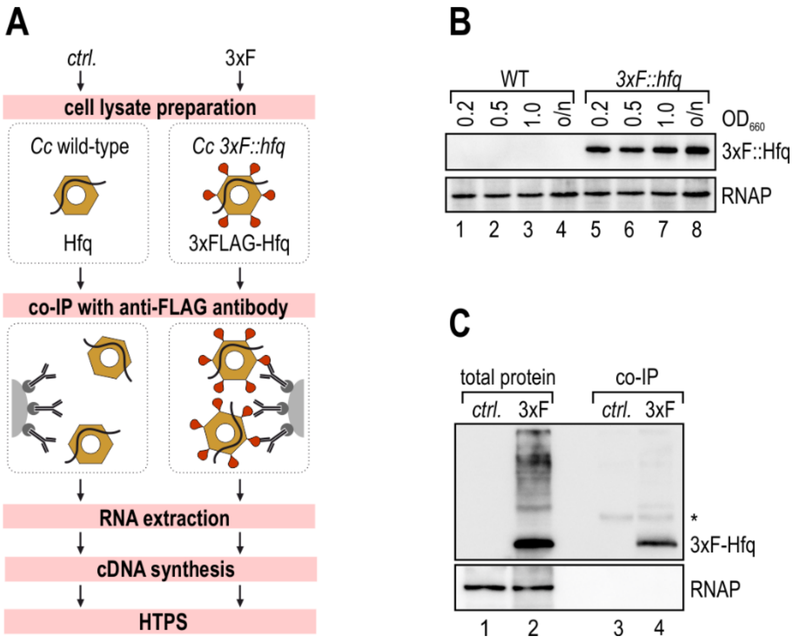

RNA co-immunoprecipitation with Hfq in *Caulobacter crescentus*.
(A) Schematic representation of the Hfq-co-IP workflow. Cell lysates were prepared from *C. crescentus* wild-type control (WT) and a strain expressing a chromosomally-encoded 3xFLAG-Hfq protein (3xF). RNA co-immunoprecipitated with the Hfq protein was purified, converted to cDNA, and analyzed by high-throughput sequencing (HTPS). (B) Western blot analysis of total protein of WT and 3xF. Samples were collected at the indicated optical density (OD_{660}) or after 24h of growth (o/n) in complex PYE medium. RNA polymerase (RNAP) served as loading control. (C) Western blot analysis of total protein and co-IP samples of WT and 3xF strains confirms pulldown of the tagged protein. RNAP served as loading control. The asterisk indicates a signal corresponding to the anti-FLAG antibody used for the co-IP.

Supplementary Figure S2

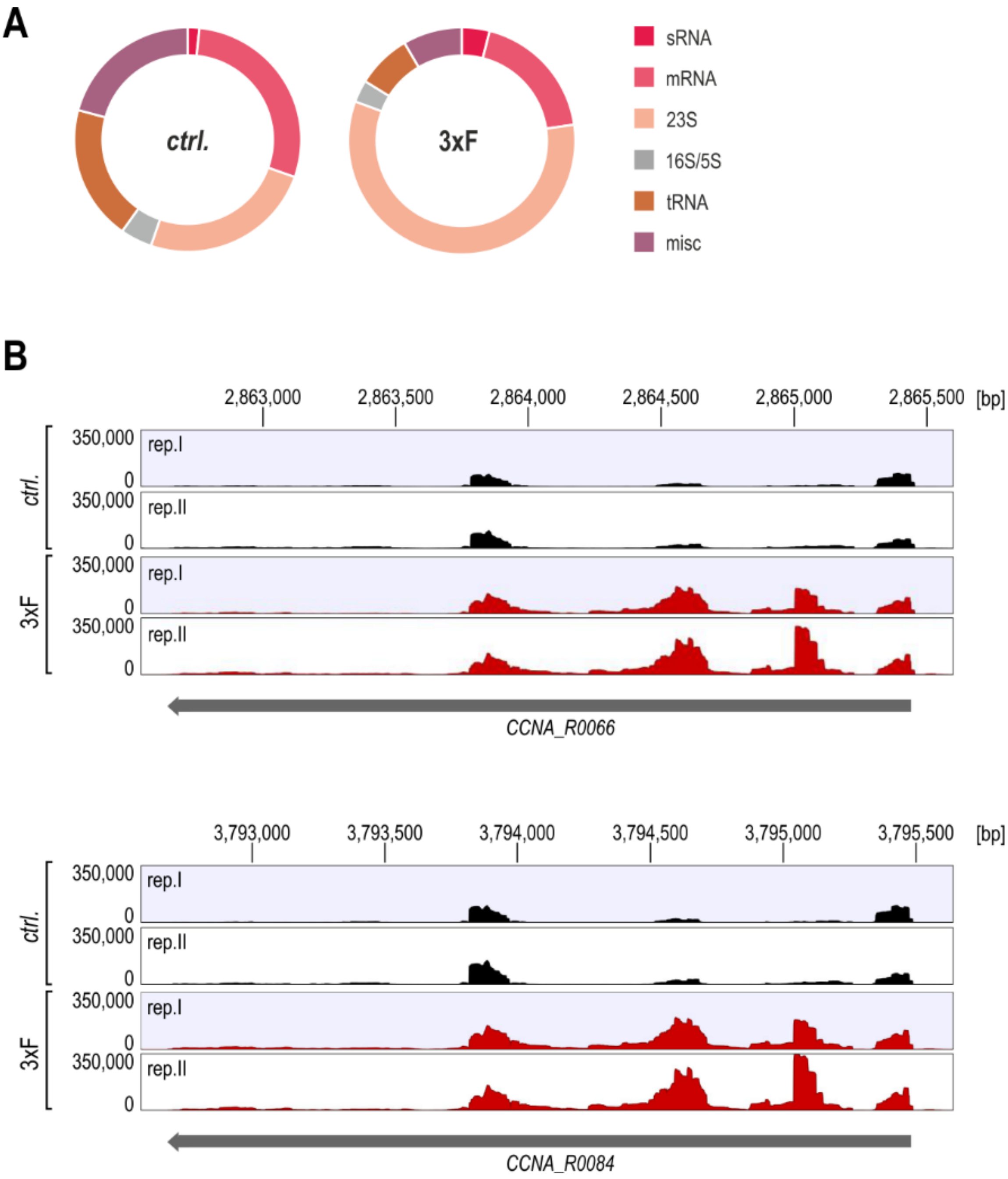

Relative read distribution RNA co-immunoprecipitation with Hfq.
(A) Circle chart illustrating the relative distribution of cDNA reads mapped to the different RNA classes of the control (*ctrl.*) and 3xFLAG-Hfq (3xF) co-IP samples. (B) Enrichment patterns of cDNA reads of the control (*ctrl.*; black) and 3xFLAG-Hfq co-IP (3xF; red) in *C. crescentus* NA1000 mapping to the 23S rRNA genes *CCNA_R0066* and *CCNA_R0084*.

Supplementary Figure S3

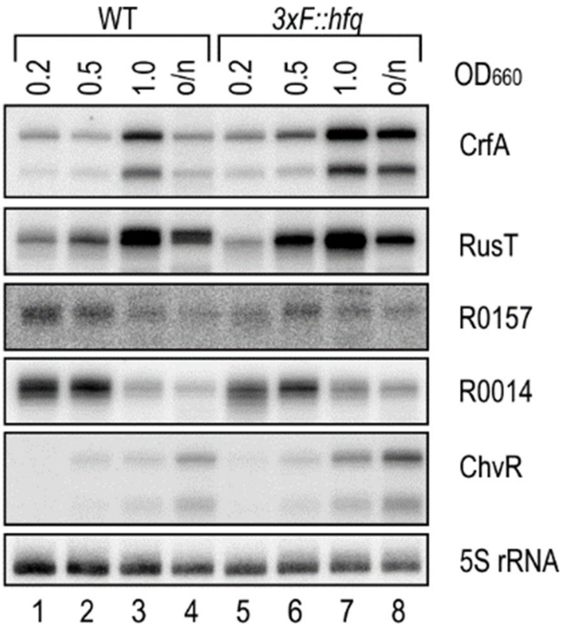

sRNA expression patterns in the presence of wild-type Hfq or 3xFLAG::Hfq protein. Expression of selected sRNAs in *C. crescentus* wild-type and an isogenic strain expressing a chromosomally-encoded 3xFLAG::Hfq protein (*3xF::hfq*) was determined by Northern blot analysis. RNA was collected at the indicated optical density (OD_{660}) or after 24h of growth (o/n) in PYE. 5S rRNA served as loading control.

Supplementary Figure S4

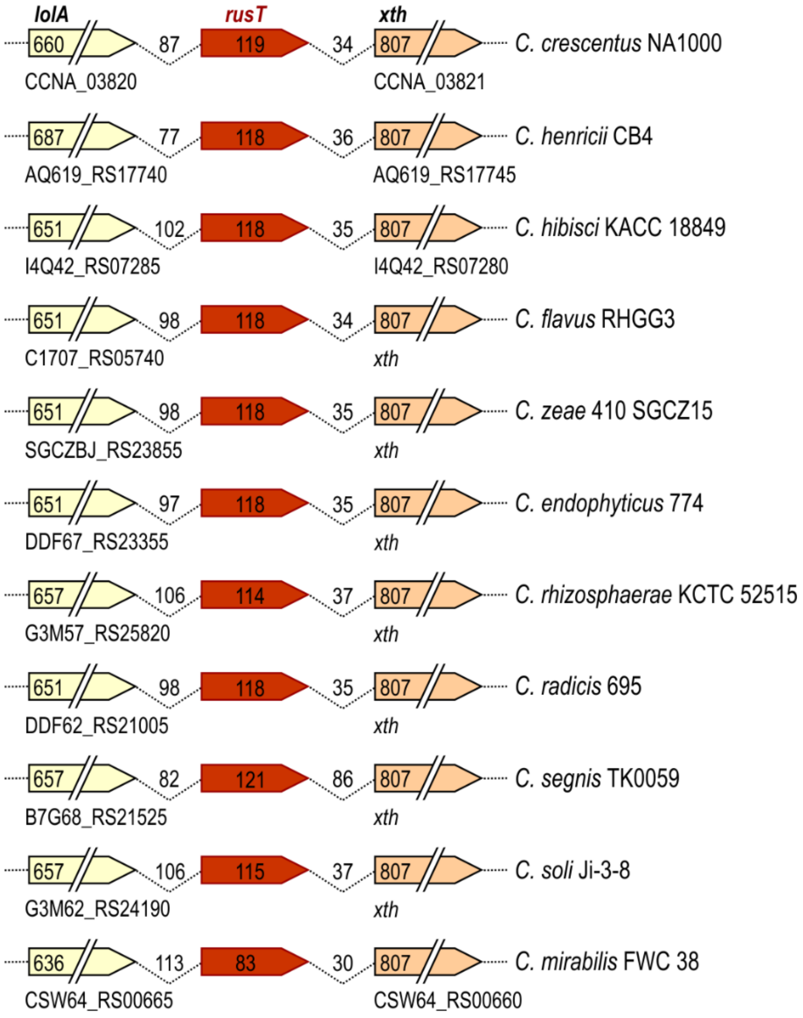

Synteny analysis of the *rusT* gene reveals conservation of the sRNA and the genomic locus within the family of *Caulobacteraceae*.

In all cases, *rusT* is positioned in the intergenic region between homologues of *CCNA_03820* (*lolA*; encoding an outer membrane lipoprotein carrier protein) and *CCNA_03821* (*xth*; encoding exodeoxyribonuclease III). Gene sizes and distances to flanking genes are indicated in bp.

Supplementary Figure S5

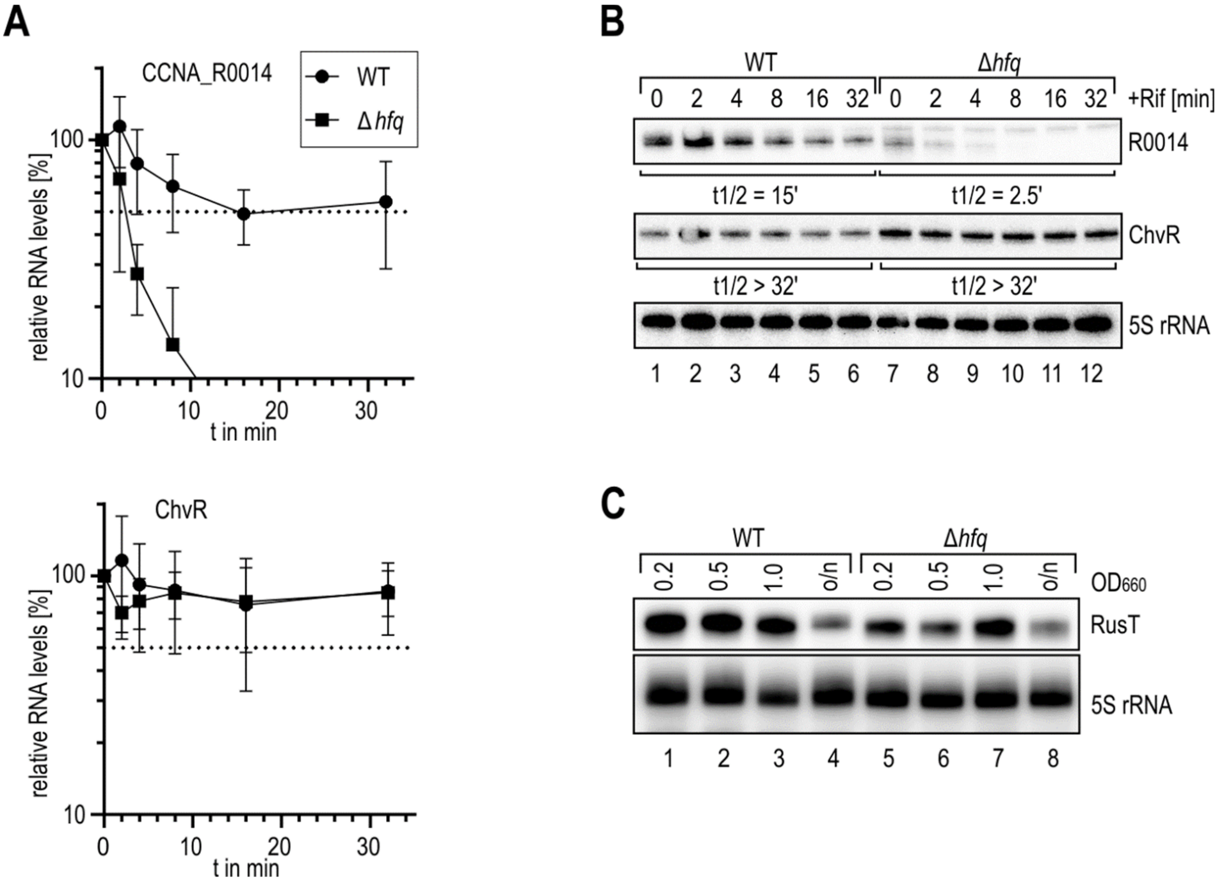

Stability of sRNAs in wild-type or Δ*hfq* cells.

(A,B) Total RNA was prepared from cells grown in minimal M2G medium to exponential phase (OD_{660} of 0.5). Transcription was inhibited by the addition of rifampicin, and RNA samples were collected at indicated time-points. Relative RNA abundance was plotted in (A) based on the quantification of signal intensities determined by Northern blot analysis as in (B). Error bars indicate the standard deviation of three biological replicates. (C) Expression of RusT in *C. crescentus* Δ*vanAB* Δ*rusT* cells carrying the expression plasmid pP_{van}-RusT. Cells were grown o/n in the presence of vanillate and RNA levels were determined at the indicated optical density (OD_{660}) or after 24 h of growth (o/n). 5S rRNA served as loading control.

Supplementary Figure S6

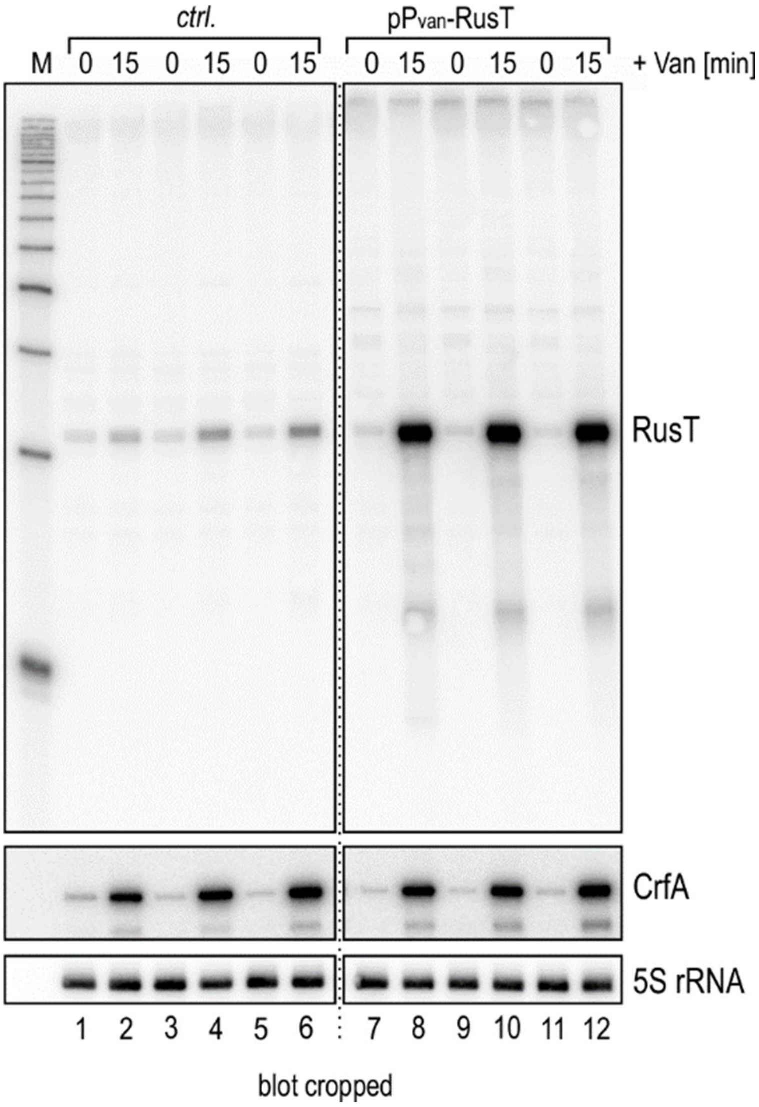

CrfA expression in response to addition of vanillate.

C. crescentus Δ*vanAB* carrying either an empty control vector (*ctrl.*; pBV-MCS6) or the expression plasmid pP_{van}-RusT were grown in biological triplicates in M2G to OD_{660} of 0.8. Total RNA was prepared from cells collected prior to and 15 min after addition of vanillate. Expression of RusT and CrfA was determined by Northern blot analysis. 5S rRNA served as loading control. The dashed line indicates that the blot has been cropped to prepare this figure.

Supplementary Figure S7

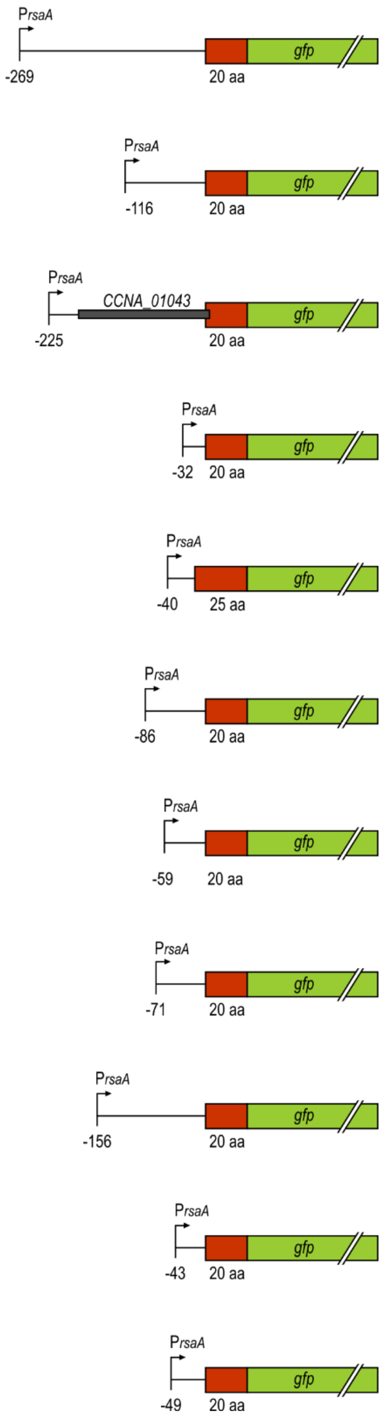

	target fusion	plasmid ID	regulation	method
A	***02895::gfp***	pKF493	yes	FI/WB
B	***03263::gfp***	pKF494	yes	WB
C	***01043::gfp***	pKF495	yes	WB
D	***02400::gfp***	pKF496	yes	WB
E	***ompW::gfp***	pKF497	yes	FI
F	***01738::gfp***	pKF498	nd	FI/WB
G	***00210::gfp***	pKF501	yes	FI/WB
H	***02380::gfp***	pKF499	yes	WB
I	***03181::gfp***	pKF500	yes	FI
J	***01043::gfp***	pKF502	no	FI
K	***03248::gfp***	pKF503	yes	WB

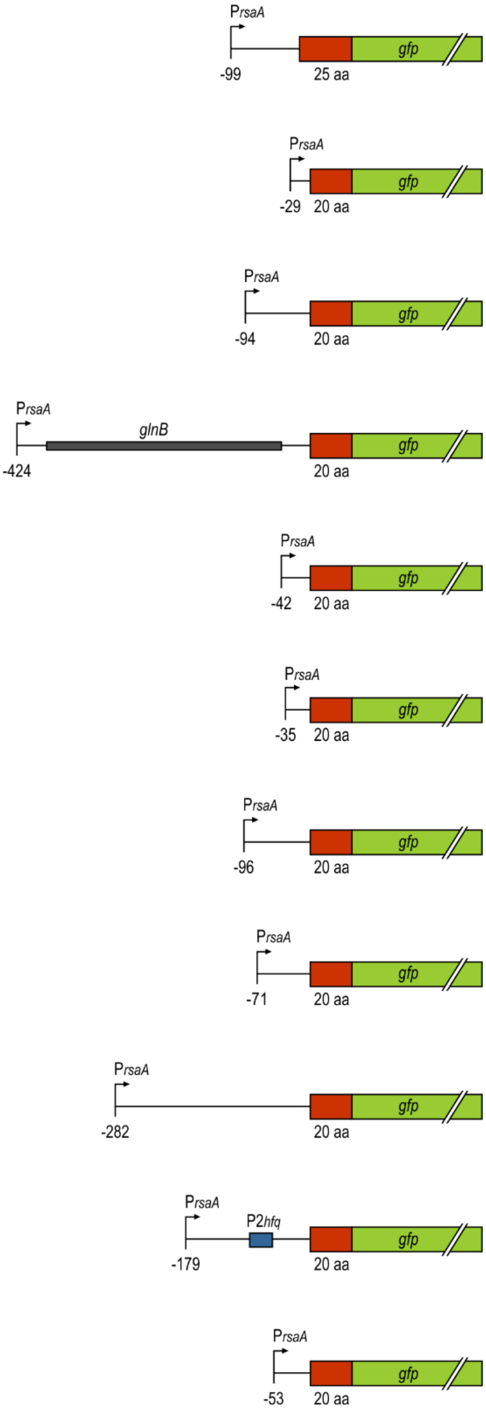

	target fusion	plasmid ID	regulation	method
L	***02741::gfp***	pKF544	yes	WB
M	***03774::gfp***	pKF545	yes	FI
N	***00338::gfp***	pKF546	nd	FI/WB
O	***glnBA::gfp***	pKF547	yes	FI
P	***glnB::gfp***	pKF548	no	FI
Q	***02342::gfp***	pKF549	yes	FI
R	***03294::gfp***	pKF504	yes	FI
S	***02380::gfp***	pKF550	no	FI
T	***03360::gfp***	pKF551	yes	FI
U	***hfq_P1::gfp***	pKF506	yes	FI
V	***hfq_P2::gfp***	pKF505	no	FI

Design of RusT target candidate reporter fusions.
Schematic representation of the translational reporter fusions of RusT target candidates. For each target, the 5′ untranslated region plus the first 20-25 codons (marked in red) are fused to *gfp* (marked in green) and expressed under control of the constitutive P_{rsaA} promoter. The position of the TSS relative to the start codon is indicated in nts. Regulation by RusT was evaluated by determination of GFP production of the reporter by fluorescence intensity (FI) measurements or Western blot analysis (WB).

Supplementary Figure S8

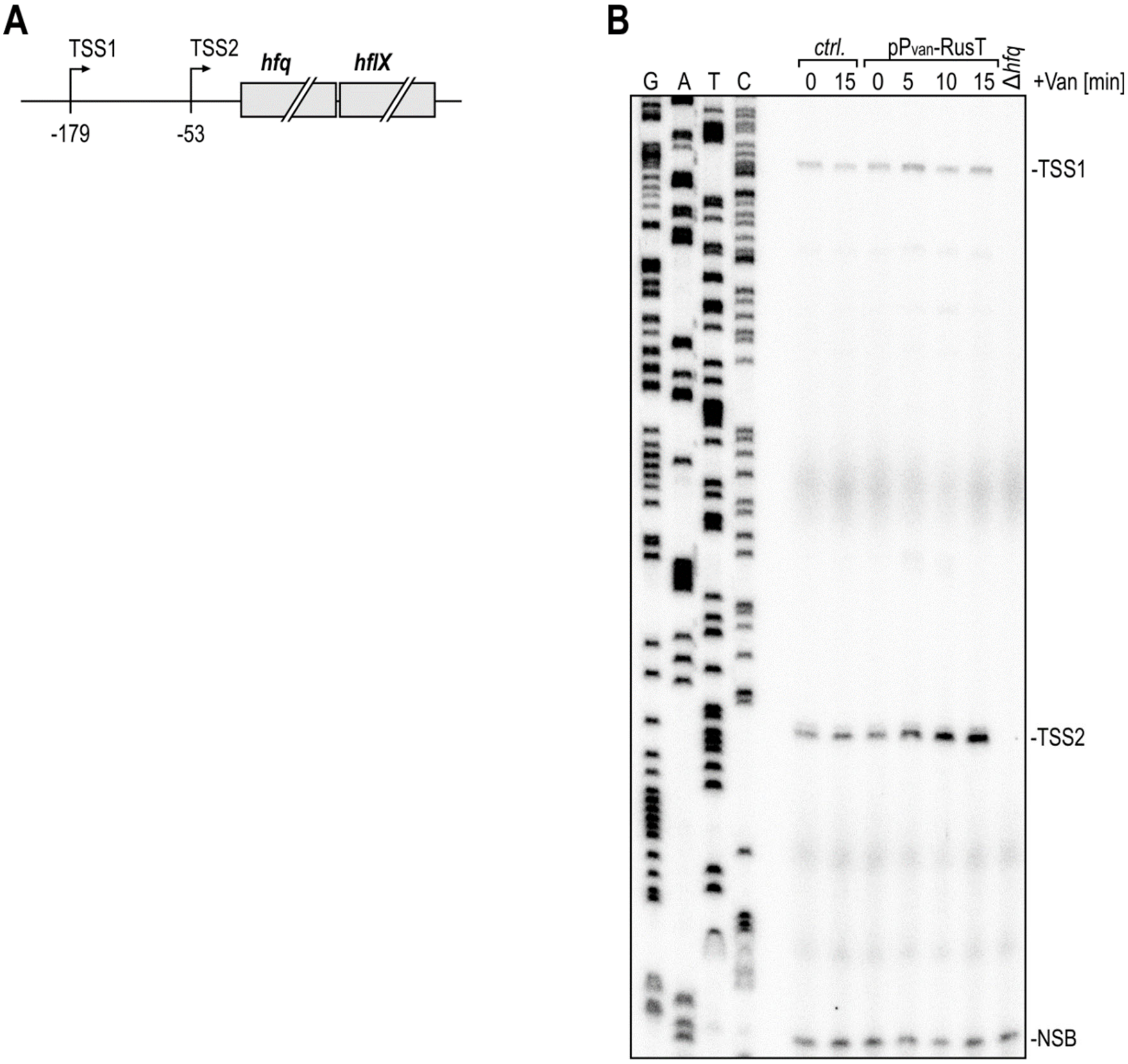

Effect of RusT overexpression on *hfq* mRNA isoforms.

(A) Schematic representation of the *hfq-hflX* locus in *C. crescentus.* (B) *C. crescentus* Δ*vanAB* carrying either an empty control vector (pBV-MCS6; *ctrl.*) or the expression plasmid pP_{van}-RusT were grown in M2G to an OD_{660} of 0.8. Total RNA was prepared from cells collected prior to and at indicated time-points after addition of vanillate (compare Fig. 5A). Size and abundance of the different isoforms of the *hfq-hflX* mRNAs transcribed from TSS 1 and TSS 2 were resolved by primer extension analysis. RNA collected from an *hfq* deletion strain was included to verify specificity of the oligo (NSB: non-specific band).

Supplementary Figure S9

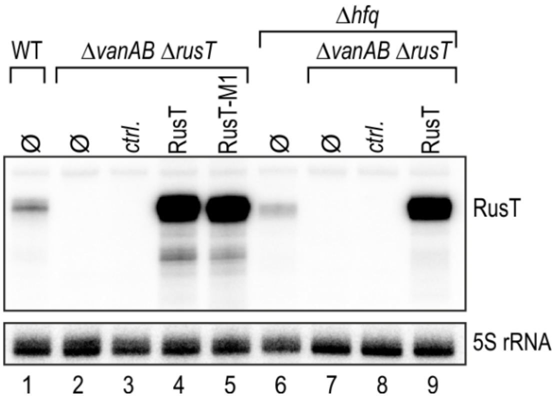

Expression of RusT variants.

Expression of RusT in *C. crescentus* wild-type, Δ*vanAB* Δ*rusT*, Δ*hfq* and Δ*hfq* Δ*vanAB* Δ*rusT* cells carrying either no plasmid (Ø), empty control plasmid pBV-MCS6 (*ctrl.*), pP_{van}-RusT or pP_{van}-RusT-M1. RNA was extracted from cells grown in PYE in the presence of vanillate to OD_{660} of ~1.0 and subjected to Northern blot analysis. 5S rRNA served as loading control.

Supplementary Figure S10

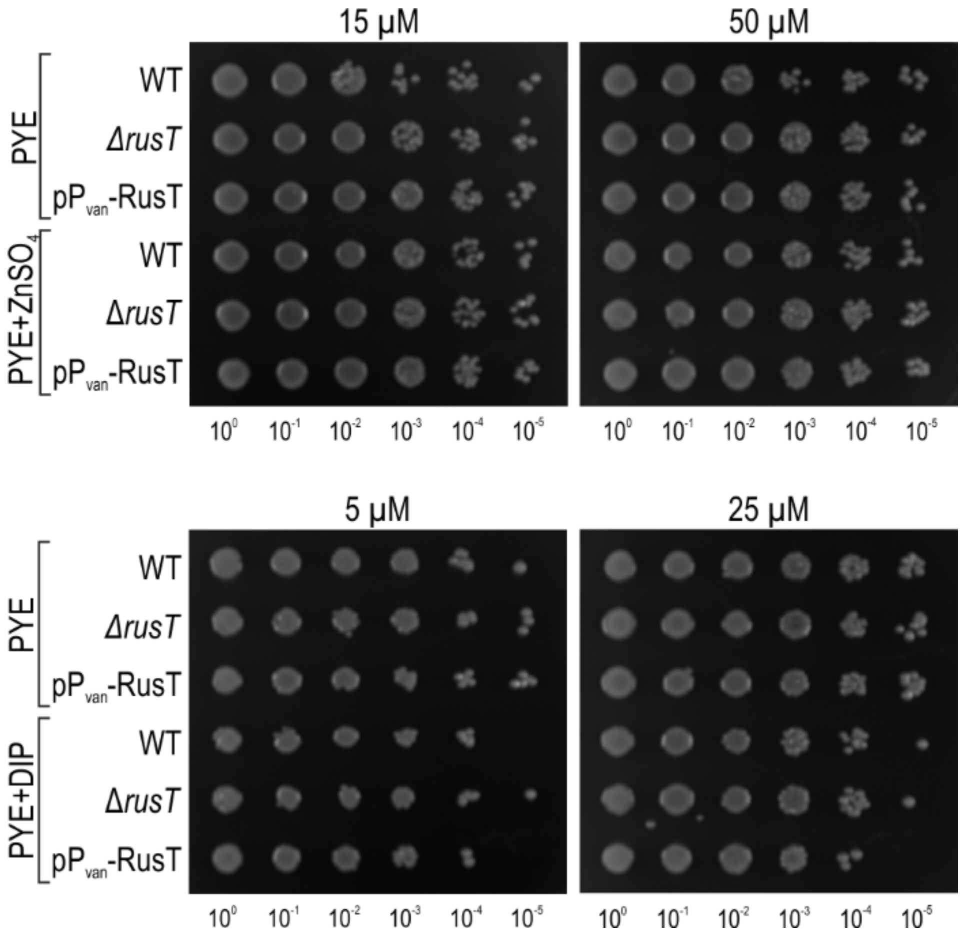

Fitness of *rusT* mutants.

Efficiency of plating of *C. crescentus* wild-type, Δ*rusT* or RusT overexpressing (pP$_{van}$-RusT in Δ*vanAB*) cells on PYE plates after 7 h of growth in PYE or PYE supplemented with the indicated concentration of $ZnSO_4$ or DIP, respectively. For RusT overexpression, cultures of Δ*vanAB* cells carrying pP$_{van}$-RusT were additionally supplemented with 0.5 mM vanillate. Spots are 10-fold serial dilutions starting from OD_{660} of 0.2.

SUPPORTING METHODOLOGY

Plasmid construction

Plasmids for allelic replacements were constructed by fusing flanking fragments of genes *rusT* (f1: KFO-0741/KFO-0742, f2: KFO-0743/KFO-0744; pKF511-8) or *CCNA_00210* (f1: KFO-2481/KFO-2482, f2: KFO-2483/2484; pKF889-5) with linearized plasmid pNPTS138 (KFO-0059/KFO-0060) at the multiple cloning site using Gibson assembly according to the manufacturer's recommendation (NEB, #E2611).

The transcriptional reporter plasmid pKF507-1 was constructed by inserting the *rusT* promotor region (spanning -87 to +8 of the *rusT* gene relative to the TSS; amplification by KFO-0699/KFO-0700; ClaI/NheI restricted) into the equally restricted pKF383-7 backbone ((1); amplified by PCR via KFO-0697/KFO-0698). To obtain the transcriptional reporter plasmid pKF776-1, a *nptII* fragment (amplified from pRL27 (2) by PCR via KFO-1785/KFO-1786; NdeI/SalI restricted) was inserted into pKF507-1 (XhoI/NdeI restricted), replacing the *lacZ* gene.

Plasmid pVan-RusT (pKF482-1) was constructed by ligation of the *rusT* fragment (PCR-amplified from *C. crescentus* gDNA using KFO-0684/KFO-0631; XbaI restricted) to the pBVMCS-6 backbone ((3); PCR-amplified with KFO-0056/KFO-0144 at the +1 site of the vanillate-inducible promoter; XbaI restriction).

To construct post-transcriptional *gfp* reporter fusions under the control of the constitutive *rsaA* promoter, the 5' UTR and first 20-25 codons of each target gene (inserts amplified from gDNA; see Table 2 and Figure S7 for details) were cloned into KpnI and EcoRI or MfeI (for pKF494-2) restriction sites of pKF385-2 as in (1).

Single nucleotide mutations were introduced by PCR amplification of the original plasmids, DpnI digestion of template DNA, and self-ligation of purified PCR products. Plasmid pKF482-1 served as a template for PCR amplification with primer pairs KFO-0750/KFO-0751 (pVan-RusT-M1; pKF512-2) and KFO-1575/KFO-1576 (pVan-RusT-SL3mut; pKF732-3). Correspondingly, reporter fusion plasmids served as templates for PCR amplification with primer pairs as follows: plasmid pKF493-2 with KFO-1641/KFO-1642 (pP*rsaA-CCNA_02895-M1::gfp*; pKF740-1), pKF497-2 with KFO-0983/KFO-0984 (pP*rsaA-ompW-M1::gfp*; pKF563-1), pKF501-1 with KFO-0989/KFO-0990 (pP*rsaA-CCNA_00210-M1::gfp*; pKF566-1).

Construction of bacterial strains and growth conditions

Genomic deletions and insertions in *C. crescentus* were obtained using a two-step recombination procedure (4). Chromosomal mutations were transferred by phage Cr30 transduction following standard protocols (5).

C. crescentus was cultivated aerobically at 30°C in either complex PYE medium, or in minimal M2 salts containing 0.2% glucose, 0.3% maltose or 0.2% xylose as carbon source (82). Where appropriate, media were supplemented with antibiotics at the following concentrations (liquid/solid): kanamycin (5/25 µg/mL); chloramphenicol (2/1 µg/mL); oxytetracycline (2/1 µg/mL); gentamycin (1/1 µg/mL). Expression from the *vanAB* promotor was induced by addition of a final concentration of 0.5 mM vanillate to cultures.

To test expression of RusT under different conditions, *C. crescentus* wild-type was cultivated in PYE to OD_{660} of 0.4. The culture was split and either resuspended in PYE at a pH of 5.5 or pH 8.5, respectively, subjected to a temperature shift (20 °C, 37 °C or 42 °C), or to chemical stress by addition of 85 mM NaCl, 40 mM KCl, 150 mM sucrose, 0.2% xylose, 0.2% glucose, 200 µM 2,2'-dipyridyl (DIP), 75 µM $ZnSO_4$, 30 µM $CuSO_4$, 10 mM H_2O_2 or 10 mM paraquat, respectively.

To analyse RNA stability, cells grown in M2G were treated with rifampicin (200 µg/mL) at an OD_{660} of 0.5 to terminate transcription. RNA samples were collected at the indicated time points and transcript levels were determined by Northern Blot analysis.
Escherichia coli strains were grown aerobically at 37 °C in LB broth and supplemented with kanamycin (50 µg/mL), chloramphenicol (20 µg/mL) or tetracyclin (12 µg/ml) where appropriate. For conjugation, *E. coli* WM3064 was grown in LB with antibiotics at 30 °C under agitation in media supplemented with 0.3 mM meso-diaminopimelic acid (mDAP) to enable growth for WM3064 derivatives (6).

Hfq co-IP
Duplicates of *C. crescentus* wild-type and cells expressing 3XFLAG-Hfq (KFS-0344) were grown in PYE medium to OD_{660} of 1. Expression of the tagged protein under the tested condition was confirmed by immunoblot analysis (Fig. S1B). Cell pellets corresponding to 50 OD_{660} were collected and subjected to immunoprecipitation as described previously (7). cDNA libraries were prepared using the NEBNext Small RNA Library Prep Set for Illumina (NEB; E7300) according to the manufacturer's instructions. cDNA libraries were pooled and sequenced using an Illumina MiSeq system in paired end mode. Demultiplexed raw reads were imported into the CLC Genomics Workbench (Qiagen) and subjected to quality control and adaptor trimming. The trimmed reads were mapped to the *Caulobacter crescentus* NA1000 reference genome (NC_011916) with standard parameter settings. The dataset has been deposited at the National Center for Biotechnology Information's Gene Expression Omnibus (GEO) repository (8), and is available via the GEO accession GSE148206.

Transcriptome analysis using RNA-seq
C. crescentus (Δ*vanAB*) cells carrying either the control vector pBV-MCS6 or a plasmid to express RusT under control of the vanillate-inducible promoter (pVan-RusT; pKF482-1) were grown in triplicates in M2 medium supplemented with glucose. RNA samples were collected prior to and 15 min after addition of vanillate to the culture at OD_{660} of 0.8. Total RNA was purified, digested with DNase I, and RNA integrity was confirmed using a Bioanalyzer (Agilent). cDNA libraries were prepared using the NEBNext Ultra II Directional RNA Library Prep Kit for Illumina (NEB, #E7760) and sequenced using a HiSeq 1500 System in single-read mode. The read files in FASTQ format were imported into CLC Genomics Workbench (Qiagen), trimmed and mapped to the *Caulobacter crescentus* NA1000 reference genome (NC_011916) using the "RNA-Seq Analysis" tool with standard parameters. Read counts were normalized (CPM) and transformed (log_2). Differential expression was tested using the built in tool corresponding to edgeR in exact mode with tagwise dispersions. Genes with a fold change ≥ 2.0 and a FDR-adjusted *p*-value ≤ 0.05 were considered as differentially expressed. The dataset has been deposited at the GEO repository, and is available via the GEO accession GSE148208.

Chromatin Immunoprecipitation coupled to deep sequencing (ChIP-seq)
Culture of exponentially growing *C. crescentus* CB15 *ntrX::ntrX-HA* (OD_{660} of 0.5, 80 ml of culture in PYE) was supplemented with 10 µM sodium phosphate buffer (pH 7.6) and then treated with formaldehyde (1% final concentration) at RT for 10 min to achieve crosslinking. Subsequently, the culture was incubated for an additional 30 min on ice and washed three times in phosphate buffered saline (PBS, pH 7.4). The resulting cell pellet was stored at -80 °C. After resuspension of the cells in TES buffer (10 mM Tris-HCl pH 7.5, 1 mM EDTA, 100 mM NaCl) containing 10 mM of DTT, the cell resuspension was incubated in the presence of Ready-Lyse lysozyme solution (Lucigen, #186002) for 10 min at 37 °C, according to the manufacturer's instructions. The lysate was sonicated (Bioruptor Pico) at 4 °C using 15 bursts of 30 sec to shear DNA fragments to an average length of 0.3–0.5 kbp and cleared by

centrifugation at 14,000 rpm for 2 min at 4 °C. The volume of the lysate was then adjusted to 1 ml using ChIP buffer (0.01% SDS, 1.1% Triton X-84 100, 1.2 mM EDTA, 16.7 mM Tris-HCl [pH 8.1], 167 mM NaCl) containing protease inhibitors (Roche) and pre-cleared with 80 µl of Protein-A agarose (Roche) and 100 µg BSA. Five percent of the pre-cleared lysate was kept as total input sample (negative ChIP control sample). The rest of the pre-cleared lysate was then incubated overnight at 4°C with a monoclonal rabbit Anti-HA Tag antibody (Millipore, clone 114-2C-7; 1:400). The immuno-complexes were captured by incubation with Protein-A agarose beads (pre-saturated with BSA) during a 2 h incubation at 4°C and then, washed subsequently with low salt washing buffer (0.1% SDS, 1% Triton X-100, 2 mM EDTA, 20 mM Tris-HCl pH 8.1, 150 mM NaCl), with high salt washing buffer (0.1% SDS, 1% Triton X-100, 2 mM EDTA, 20 mM Tris-HCl pH 8.1, 500 mM NaCl), with LiCl washing buffer (0.25 M LiCl, 1% NP-40, 1% deoxycholate, 1 mM EDTA, 10 mM Tris-HCl pH 8.1) and finally twice with TE buffer (10 mM Tris-HCl pH 8.1, 1 mM EDTA). The immuno-complexes were eluted from the Protein-A beads with two times 250 µL elution buffer (SDS 1%, 0.1 M NaHCO3, freshly prepared) and then, just like the total input sample, incubated overnight with 300 mM NaCl at 65°C to reverse the crosslinks. The samples were then treated with 2 µg of Proteinase K for 2 h at 45°C in 40 mM EDTA and 40 mM Tris-HCl (pH 6.5). DNA was extracted using phenol:chloroform:isoamyl alcohol (25:24:1), ethanol-precipitated using 20 µg of glycogen as a carrier and resuspended in 30 µl of DNase/RNase free water.

Immunoprecipitated chromatins were used to prepare sample libraries used for deep-sequencing at Fasteris SA (Geneva, Switzerland). ChIP-Seq libraries were prepared using the DNA Sample Prep Kit (Illumina) following the manufacturers' instructions. A single-end run was performed on an Illumina Next-Generation DNA sequencing instrument (NextSeq High), 50 cycles were performed and yielded several million reads per sequenced samples. The single-end sequence reads stored in FastQ files were mapped against the genome of *C. crescentus* NA1000 (NC_011916.1) using Bowtie2 Version 2.4.5+galaxy1 available on the web-based analysis platform Galaxy (https://usegalaxy.org) to generate the standard genomic position format files (BAM). ChIP-Seq reads sequencing and alignment statistics are summarized in Supplementary Table S2. Then, BAM files were imported into SeqMonk version 1.47.2 (http://www.bioinformatics.babraham.ac.uk/projects/seqmonk/) to build ChIP-Seq normalized sequence read profiles. Briefly, the genome was subdivided into 50 bp, and for every probe, we calculated the number of reads per probe as a function of the total number of reads (per million, using the Read Count Quantitation option). Analysed data illustrated in Figure 4 are provided in Supplementary Table S2. Using the web-based analysis platform Galaxy (https://usegalaxy.org), NtrX-HA ChIP-Seq peaks were called using MACS2 Version 2.2.7.1+galaxy0 (No broad regions option) relative to the total input DNA samples. The q-value (false discovery rate, FDR) cut-off for called peaks was 0.05. Peaks were rank-ordered according to their fold-enrichment values (Supplementary Table S2, peaks with a fold-enrichment values >2 were retained for further analysis). Sequence data have been deposited to the Gene Expression Omnibus (GEO) database (GSE247928 series, accession numbers GSM7903192 and GSM7903193).

T7 transcription and 5' end labelling of RNA

RNAs were synthesized by *in vitro* transcription and 5' end-labelled as described previously (9, 10). In short, a DNA template carrying the T7 promoter was amplified by PCR (KFO-0892/KFO-0893 on gDNA for RusT; KFO-0892/KFO-0893 on pKF512-2 for RusT-M1; KFO-0351/0352 on gDNA for ChvR; KFO-0513/0514 on gDNA for R0014; KFO-0563/0564 on gDNA for R0157; KFO-0937/938 on gDNA for CrfA) and transcribed using the AmpliScribe T7-Flash transcription kit (Epicentre). Purified RNA (20 pmol) was dephosphorylated using calf intestinal alkaline phosphatase (NEB), and recovered by P:C:I extraction and ethanol precipitation. 5' end-

labelling was achieved by incubation of dephosphorylated RNA with [^{32}P]-γATP (25 µCi) and polynucleotide kinase (1 unit; NEB) for 1 h at 37°C. RNA was then purified on a denaturing 6% PAA / 7 M urea gel, eluted in RNA elution buffer (0.1 M sodium acetate, 0.1% SDS, 10 mM EDTA) at 4°C overnight and recovered by P:C:I extraction.

Primer extension analysis
For primer extension, 5 µg of RNA were denaturated in the presence of 1 pmol 5′ end-labelled primer (KFO-0966) at 70°C for 2 min and adjacently chilled on ice for 5 min. Next, the samples were mixed with the reaction mix (1X first strand buffer, 5 mM DTT, 0.5 mM each dATP, dGTP, dCTP and dTTP) at 42°C, and SuperScript III (100 U; Invitrogen) was added. cDNA synthesis was performed at 50°C for 60 min, followed by incubation at 70°C for 15 min to inactivate the enzyme. Samples were treated with RNase H (2.5 U) for 15 min at 37°C and the reaction was stopped by the addition of GLII loading buffer. Samples was separated electrophoretically together with a template-specific ladder (prepared using the SequiTherm EXCELII DNA Sequencing Kit) on a 6% sequencing gel.

Electrophoretic Mobility Shift Assays
Complex formation between sRNAs and Hfq was analysed *in vitro* using gel mobility shift assays following previously established protocols (11). In short, denatured 5’ end labelled sRNA (4 nM final concentration) was incubated with purified *C. crescentus* Hfq (lab stock; concentration as indicated in the figure legend) in the presence of 1 µg yeast RNA and 1x structure buffer (0.01 M Tris-HCl [pH 7], 0.1 M KCl, 0.01 M MgCl2) or Hfq dilution buffer (1x structure buffer, 1% glycerol, 0.1% Triton X-100) at 30°C for 15 min. Reactions were mixed with native loading buffer (50% glycerol, 0.5x TBE, 0.2% bromophenol blue) and separated by native PAGE. Gels were dried and signals visualized on a phosphor imager.

RNA structure probing
RNA structure probing and mapping of Hfq footprints was conducted as described previously (12) with some alterations. Briefly, 0.4 pmol 5’ end-labelled RNA was denatured, cooled on ice and mixed with *C. crescentus* Hfq (0.4 pmol or 2 pmol) or Hfq dilution buffer in the presence of 1X structure buffer (0.01 M Tris pH 7, 0.1 M KCl, 0.01 M $MgCl_2$) and 1 µg yeast RNA (Invitrogen, #AM7118). Upon incubation at 30°C for 10-15 min, the samples were treated with RNase T1 (0.1U; Ambion, #AM2283) for 2.5 min, with lead(II) acetate (final concentration: 5mM; Sigma-Aldrich, # 316512-5G) for 1.5 min or with RNase V1 ($2x10^{-5}$ U; Ambion, #AM2275) for 1 min, respectively. Reactions were stopped by addition of 2 vol. equiv. precipitation buffer (1 M guanidinium thiocyanate, 0.167% N-laurylsarcosine, 10 mM DTT, 83% 2-propanol) and precipitated at -20°C overnight. Pellets were washed with 70% ethanol, and dissolved in GLII loading buffer. The RNase T1 and alkaline (OH) sequencing ladders were prepared with 0.8 pmol 5’ end-labelled sRNA and stopped by addition of 1 vol. equiv. of GLII. Samples were separated by denaturing PAGE on 10% PAA / 7 M urea sequencing gels.

Bioinformatic tools
Sequence alignments were generated using MultAlin ((13); http://multalin.toulouse.inra.fr/). RNAfold (http://rna.tbi.univie.ac.at/cgi-bin/RNAWebSuite/RNAfold.cgi) was employed to predict RNA secondary structures. Predictions for RNA base-pairing interactions were determined with the RNAhybrid ((14); https://bibiserv.cebitec.uni-bielefeld.de/rnahybrid/) and IntaRNA ((15); http://rna.informatik.uni-freiburg.de/IntaRNA/) algorithms.

Table S3 Secondary structure and nucleotide composition of Hfq-associated sRNAs (≥ 3-fold enrichment)

sRNA	sequence	structure	length (in nt)	nt in structures (in %)	terminator	U-run	GC content (in %)	GC content -15 nt terminator (in %)
CCNA_R0093	gcaaggacgaaacgagcccaccagagagcucccguucuucggcguuu<br>cggggaggaaacgcccgccuagaucgaaugaucgagaaaauagccag<br>cccgucgcgauuaucccccgcggcggguuuggugu	..(((((((....((((..........)))).))))))).(((<br>(((((......))))))))...((.((((....)))).)).......<br>....(((((((((.........))))))))).......	129	53,5	yes	weak	60,5	46,7
R0199 (RusT)	gcauggcgaugggcgaccgacccgcuccucgcaaaccgugccg<br>gaaccuaucccucccggcggcggcaugagagacuagacuuucgccc<br>agaugcucgacgcaucugggcguuuuucu	.....((((.(((((.......))))).))))...((((((((<br>(.............))))).))))....(((((......))))(((((<br>((((((.....)))))))))))....	119	56,3	yes	yes	63,9	40,0
CCNA_R0157	gugaggcgccgagcgccucugcccuuccugggcguuccuccc<br>uaugacuuuguagcccggucuucauggccgggcuuucuuuuu	..(((((((...))))))).((((.....))))..........<br>............((((((((.....)))))))).........	85	44,7	yes	yes	60,0	40,0
CCNA_R0014	gugaggcggcgcugccgcuucugcccuuccugggcguuccuc<br>ccuaaugaacuggcccggcggguuucgccgggcuuuuuuuu	..((((((((...)))))))).((((.....))))........<br>............((((((((....)))))))).........	83	49,4	yes	yes	62,7	46,7
CCNA_R0188	agaaguucggccgagaucaucugcggggguggagggggcgucgg<br>ugggcugaccgacacugcagcuaccugaaaugcagagaaaaugaucc<br>gcgccggggaggacguucgcgggaauucaugggcuuuggaugcgccu<br>ugcacgcguccugacguuucgaagucgguggauuucgcgagcgugac<br>guauugcucgcaaugcgucggacuuugguaaagcgcaacucggcgcg<br>ggagccucucccgcgccguuuucuuau	.....((((((((.((((((((((((((((....((.(((((<br>(......)))))).))....))))))....))))))).....))))))<br>).)))))).....(((((((((.(((((((.(((((((((((((...<br>....))))))).........)))))).)))))))))))))))))(((<br>((((((....)))))))))).....(((.........)))..(((((((<br>((((...)))))))))))).........	258	67,1	yes	yes	60,1	46,7
CCNA_R0143	gugaggcugagagccucugcccuuccuggggcguuccucccu<br>acuuauuucagcccggcuuuucgccgggcuuuucuuu	..((((((...)))))).(((((....)))))...........<br>..........(((((((.....))))))).........	80	45,0	yes	yes	57,5	33,3
CCNA_R0097	aucaaugugaaguacugauccucccccuuagggugcuucacac<br>ccagcgccccguccgaccugugucggucggggcguuuuucc	.....(((((((((((...............))))))))))).<br>....(((((((.(((((....))))).))))))).......	84	56,0	yes	yes	59,5	60,0
CCNA_R0195	guucgcacuaaagcaccuauaaaccacagggugaacuggcccg<br>aauuuccaagccucgaaaaauccguuuacaagggggcgcccuuuauc<br>cacaagaaagcgaccgugcgcgaaguaaagaagcuuuuggagcgcua<br>gguugcuuaucggaaugucuagcgccgcagccuuaucgaggagcagg<br>uuggcaacugcgcgcgcgucaaccgacugagggucuugagucagcgg<br>aggcaugaauccaaccucauugagccccccucacggcaagccaggcu<br>cgccuuagaaauucgcgagucgcaaaauagaacauaaugcgaacucu<br>caugccguccaacccgaucgucucucaaaaucgucacccauacucaa<br>uccuuaauguucaugguuaaggaagguugaggaauuuugcgugagag<br>acagcacugcgagugcgccccuacaucucacggcgagacgaucgcug	(((((((......(((((..........)))))..(((((...<br>..((((.........))))...((((.....((((((.((.((((....<br>...))).((((((((..(((((((((......)))))....))))..<br>)))))))).((((..((....((((((((((..(((.........))<br>).)))...)))).))))..)).)))).(((((.....((.(((((..<br>..)).))).))...)))))....)))))))).))))...))))).((<br>(((............)))))..................))))))...<br>..............(((((((((.(((((((.(((.((........<br>((((((((.......)))))))).)).))).)).)))))((((((.<br>...(((((...)))))........))))))..)))))))))...	466	56,7	nd	no	53,0	nd
CCNA_R0040	aucuaaagcuaaagggcucuaggcagccgauauucggcggugu<br>gcgaaacgucacaucgccagggggggcgggcgucuaccaacgacuug<br>ucugcucuugugggagucggaccgguggggcgaugggcuccgcgugc<br>aacggccgucagagcugaagacgcgaaccgaucgggggcgggcggug<br>gagaccugaaggggguuccgccguccucuuc	(((....((.....((((((.(((.((((......((((((((<br>(((...)).)))))))))((((.((((((((((......))).))))<br>))).))))...((.........)).(((((((.....))))))).....<br>..)))).)))))))))......))......)).......((((((((<br>((.((((....)))))))))))))).....	214	62,6	yes	weak	64,5	73,3
CCNA_R0133	agcccuuucgggcguuuccucccaaugacucggccgcccucuu<br>cuccuggagggcggccguucuuuu	.((((....))))................((((((((((((.<br>.....)))))))))))).......	67	47,8	yes	yes	62,7	46,7

CCNA_R0025	uuuuuaugaaccggaggcguuaauccgggcuuguaauguuuau<br>ggcaggggcggggaucuucuuucuugauguggagcuagugucggaga<br>acucgauaaauuguuagauaacuuccacucuguuugauggucgcggu<br>gucaucguugauauuuugaccgcugcgucaggaaucugaggcgcgcu<br>ccgcaauagcguaaguugugcucugaauucacgacuaagugucggau<br>ucuuggagcagaaagguugcgaaccggcgccgguuaagauaugaaga<br>gagggucuacccacaagacuucccauuagccccgccggagaccgcgu<br>caccggcggggc	...((((((.(((((........)))))..)))))).....((<br>((.(((...((((((((((..((((.((((..((((.(((((((..(<br>(((.........((((((((.........))))))))(((((.(((<br>((((....))))))).))))).((((.(((((((((((.(((((((<br>.......))))).(((((((.........)))))))..)).))))))<br>))))))).))))...)))).....))))))).))))...)))).))))<br>)))))))..)))......))).))))..((((((((((.(((...))<br>).))))))))))	337	67,7	yes	no	51,9	nd
CCNA_R0127	cuccaccgcccaggucacacuggcggcucggagccugccgguu<br>uccccggcggcgaaguagugagcguccgguaggcggcugcacgccuc<br>ccgccguucccugcgucucuuccccucucaaggagcgucgccauccg<br>cgacgcucccuuuuuuu	((((.((((((.((......)))))))...)))).(((((((..<br>...))))))).((.((((.(((((..(((.((((((......)))))).<br>)))).))))).)))).))..............((((((((((.....)<br>))))))))))........	154	61,0	yes	yes	68,2	53,3
CCNA_R0098	uucucgcagggcgcgggaucgaucgcugggcacuggaugucga<br>gcgucuguccgguuuccgcccgcuucugccucccgaauggucggcga<br>acggacgcgaggacgacuagggauuuucgccuuuggcgguuccuggg<br>cacagacggcgggccuguccgcccggucgguucgccggccuuggcag<br>gcgcuagagcuuuu	........(((((.(((((((((((((.((((.....)))).)<br>)))...)).))))))))))))((((.(((((.((((..(((((((((<br>((.(((.((.((..(((.(((....((((((((((((.........)<br>).)))).)))))))))))))..))))))).)))))))))))))))).))<br>)))...))))....	198	71,7	nd	yes	66,7	nd
CCNA_R0156	gauguagaagaggcagccugccuuggcggucucgcccuugguc<br>cgaagcgccucauagaucguccggguuuccgaguggcuuggcgcguc<br>cgacggauagcgcucggggcggacggagaacugcuggaucgcagagg<br>ucucaaagcccaugccgaccaaggccuuggcgacgucucggcgagcg<br>uugucgaaggcuucgguaggacugccagacuccgccugaaagucgcc<br>ugcgaugacgaccgcgcuccaguucgagaacggcccgccggcgaagg	(((.((...(((((.((..(((..((((....))))...))))).<br>....))))))).)).)))...(((((.((((..((.((((((((((((<br>(...)))).)))).)))).))..))))...((((......)))).((<br>(((...((.((.((((((....((((((((((((.(((...)))))<br>))))).)))))))))))))))).))...)))))..))))))....(((((<br>.(((.......(((..(((......)))..)))..))).))))))...	278	65,5	nd	no	65,1	nd
CCNA_R0016	ccccuucaccaccccaccggugaagcaacucgggcggcgaccu<br>uuucgggggagggccgcccgcccuuac	...((((((((........))))))))).....((((((((..(((<br>(((...))))))))))))))).......	70	60,0	yes	no	71,4	53,3
CCNA_R0184	cuuucggggcgcguccgcuccgucccucuggccgcguuccgag<br>aagagagcccguccggucuccccaugaccgggcgggcuuuuucgu	.(((((((((((((.(((..........))).)))))))))))<br>)....((((((((((((.......))))))))))))........	88	63,6	yes	yes	69,3	53,3
CCNA_R0175	gcguacuuaggcucgcccgcgcagcccugcuuuuccgggcugu<br>cacggcguugcuaggcgcgcuucccucccccgggagcgcgccgauuu<br>uuu	.........(((.((((...((((((((.........))))))))<br>...))))..))).((((((((((((.......))))))))))))).....<br>...	93	53,8	yes	yes	67,7	60,0

Supplementary Table S4 – Oligonucleotides

oligo ID	sequence 5’ to 3’	description
KFO-0056	AAGCTTGATATCGAATTCCTGCA	amplification of pBVMCS-6
KFO-0059	CGAATTCGTGGATCCAGATATC	amplification of pNPTs138
KFO-0060	CTTCGGCCGTGACGCGTCT	amplification of pNPTs138
KFO-0113	CAGGGGGACTTAACGACCGAGTTC	oligo probe for 5S ribosomal RNA
KFO-0144	GGATCCAATCTTGATCGTAAT	amplification of pBVMCS-6
KFO-0221	ATGGCCCTCCGGAGACTTG	oligo probe for ChvR
KFO-0351	GTTTTTTTTTTAATACGACTCACTATAGGCGGG GCCTACATGTCG	amplification of ChvR DNA template for T7 *in vitro* transcription
KFO-0352	GTTTTTTTTTTAATACGACTCACTATAGGCATGT CGCGCAAGTCTCC	amplification of ChvR DNA template for T7 *in vitro* transcription
KFO-0359	ACCCGCCAGGTGAACAGTC	sequencing of plasmids with backbone pGFPC-2
KFO-0513	GTTTTTTTTTTAATACGACTCACTATAGGTGAG GCGGCGCTGCC	amplification of R0014 DNA template for T7 *in vitro* transcription
KFO-0514	AAAAAAAAGCCCGGCGAAACCG	amplification of R0014 DNA template for T7 *in vitro* transcription
KFO-0554	GTCTAGTCTCTCATGCCGC	oligo probe for CCNA_R0199 RNA
KFO-0563	GTTTTTTTTTTAATACGACTCACTATAGGTGAG GCGCCGAGCGC	amplification of R0157 DNA template for T7 *in vitro* transcription
KFO-0564	AAAAAGAAAGCCCGGCCATG	amplification of R0157 DNA template for T7 *in vitro* transcription
KFO-0577	GGGCTACAAAGTCATAGGGAG	oligo probe for CCNA_R0157 RNA
KFO-0579	GGGCCAGTTCATTAGGGAGG	oligo probe for CCNA_R0014 RNA
KFO-0580	GACCATGATTAGGCGAAGCTACGT	sequencing of plasmids with backbone pNTPS138
KFO-0581	TGTGCTGCAAGGCGATTAAGTTGG	sequencing of plasmids with backbone pNTPS138
KFO-0630	GCATGGCGATGGGCGACC	construction of plasmid pKF482-1
KFO-0631	GTTTTTCTAGAGCCCCGCTCTATCGCGCT	construction of plasmid pKF482-1
KFO-0697	ACGCTCATCGATAATTTCAC	construction of plasmid pKF507-1
KFO-0698	GCATGGCGGGTACCCACGATGCGAGGAAACG	construction of plasmid pKF507-1
KFO-0699	CGTGGGTACCCGCCATGCGTGTTAAATATG	construction of plasmid pKF507-1
KFO-0700	GTTTTGCTAGCGTCGGCGGATTTGTGACA	construction of plasmid pKF507-1
KFO-0713	CTTTTGAATTCACCGGACGTTCAGAATCCG	construction of plasmid pKF493-2
KFO-0714	CTTTTGGTACCCAGTCGCGTCGTGAAGGAA	construction of plasmid pKF493-2
KFO-0715	CTTTTCAATTGATGCTGCGAGGGGCTCGC	construction of plasmid pKF494-2
KFO-0716	CTTTTGGTACCGATCGCGACCGCCGAAGC	construction of plasmid pKF494-2
KFO-0717	CTTTTGAATTCTGCAACGCGCGGGATGCAA	construction of plasmid pKF495-1
KFO-0718	CTTTTGGTACCGGCGGGCGAAGCGATGG	construction of plasmid pKF495-1
KFO-0719	CTTTTGAATTCGAACGCGACGATAGATCGC	construction of plasmid pKF496-2
KFO-0720	CTTTTGGTACCCGCGGCGATGAAATTCGC	construction of plasmid pKF496-2
KFO-0721	CTTTTGAATTCGCGACAAGAAACAAGACGTC	construction of plasmid pKF497-2
KFO-0722	CTTTTGGTACCCGTAAAGTCTTGAGCCTGG	construction of plasmid pKF497-2
KFO-0723	CTTTTGAATTCGACCCATGTTCTCGATGCG	construction of plasmid pKF498-1
KFO-0724	CTTTTGGTACCGGCCATGGTCGCGACGCT	construction of plasmid pKF498-1
KFO-0725	CTTTTGAATTCACCTGTTTTGGGGCCTTGTG	construction of plasmid pKF499-1
KFO-0726	CTTTTGGTACCGAGGTCGGCCTGGGTCAC	construction of plasmid pKF499-1
KFO-0727	CTTTTGAATTCGTTCCGGGCGATTTTTTGCG	construction of plasmid pKF500-2
KFO-0728	CTTTTGGTACCGATCTCACCGATCTCGTACAG	construction of plasmid pKF500-2
KFO-0729	CTTTTGAATTCAAAGGCACGAAACTATGCGA	construction of plasmid pKF501-1

KFO-0730	CTTTTGGTACCGGATGCCGAAAGGGCGC	construction of plasmid pKF501-1
KFO-0731	CTTTTGAATTCTGCAACGCGCGGGATGCA	construction of plasmid pKF502-2
KFO-0732	CTTTTGGTACCGAGCGCACCGTTCAAAACG	construction of plasmid pKF502-2
KFO-0733	CTTTTGAATTCAACAAACACCCAAAAGTATA	construction of plasmid pKF503-2
KFO-0734	CTTTTGGTACCATTCGCGGACGAAAACAT	construction of plasmid pKF503-2
KFO-0735	CTTTTGAATTCGCGCAGGGTTGGGTAAAG	construction of plasmid pKF504-1
KFO-0736	CTTTTGGTACCGAAGCTATCGATGCGCTTC	construction of plasmid pKF504-1
KFO-0737	CTTTTGAATTCGGCGCGCCAGAAAGCCGT	construction of plasmid pKF505-1
KFO-0738	CTTTTGGTACCCGACTTGCGAACGCTGTTCA	construction of plasmid pKF505-1 and 506-1
KFO-0739	CTTTTGAATTCGTTTGTGTGGGGGGCGTG	construction of plasmid pKF506-1
KFO-0741	GATATCTGGATCCACGAATTCGGCAAGGCGCG GTTCGCCT	construction of plasmid pKF511-8
KFO-0742	GCGCTTTTTGGGGACCGGAAGTGTTAAATATG CCACGTCCT	construction of plasmid pKF511-8
KFO-0743	AGGACGTGGCATATTTAACACTTCCGGTCCCC AAAAAGCGC	construction of plasmid pKF511-8
KFO-0744	AGACGCGTCACGGCCGAAGCCGGCGCGATAT TGAGGTC	construction of plasmid pKF511-8
KFO-0788	CCTATCGCCTCCCGGCGGCGGCAT	construction of plasmid pKF518-2
KFO-0789	GGGAGGCGATAGGTTCCGGCACGGTTTG	construction of plasmid pKF518-2
KFO-0790	GGAGGTCGATTAAATGGGCACGGCCAACA	construction of plasmid pKF514-1
KFO-0892	GTTTTTTTTTTAATACGACTCACTATAGGCATG GCGATGGGCGAC	amplification of RusT DNA template for T7 *in vitro* transcription
KFO-0893	AGAAAAACGCCCAGATGCGT	amplification of RusT DNA template for T7 *in vitro* transcription
KFO-0898	GGCCAGCGAGCTAACGAGAC	nested arbitrary primer for mapping of MAR2xT7 insertion sites
KFO-0899	GGCCAGCGAGCTAACGAGACNNNNGTTGC	arbitrary primer for mapping of MAR2xT7 insertion sites
KFO-0901	GGCCAGCGAGCTAACGAGACNNNNAGTAC	arbitrary primer for mapping of MAR2xT7 insertion sites
KFO-0922	CTTTTGAATTCGTGCCTCCAACTAATCAGTC	construction of plasmid pKF544-1
KFO-0923	CTTTTGGTACCCTCTTGAGCCGAGGCGG	construction of plasmid pKF544-1
KFO-0924	CTTTTGAATTCAAAGCACAAAAGACTGCAGG	construction of plasmid pKF545-1
KFO-0925	CTTTTGGTACCGTTGGAAGCGGGCATATAG	construction of plasmid pKF545-1
KFO-0926	CTTTTGAATTCAATTCGCTGTTCGATGAATTG	construction of plasmid pKF546-1
KFO-0927	CTTTTGGTACCGCCGGCGATCGTGCTC	construction of plasmid pKF546-1
KFO-0928	CTTTTGAATTCAGAGAAGGCGGTTCGCCG	construction of plasmid pKF547-1 and pKF548-1
KFO-0929	CTTTTGGTACCGTCGACGTACTTCACGTCCT	construction of plasmid pKF547-1
KFO-0930	CTTTTGGTACCCAGGGCCTCCTTCACCTC	construction of plasmids pKF548-1
KFO-0931	CTTTTGAATTCAACTAAGAGAACAAAGTCATAC	construction of plasmid pKF549-1
KFO-0932	CTTTTGGTACCCATGTCGAGCTCGGCTTG	construction of plasmid pKF549-1
KFO-0933	CTTTTGAATTCACCTGTTTTGGGGCCTTGT	construction of plasmid pKF550-3
KFO-0934	CTTTTGGTACCGAGGTCGGCCTGGGTCA	construction of plasmid pKF550-3
KFO-0935	CTTTTGAATTCCTCTGAACCACGCAGGCG	construction of plasmid pKF551-1
KFO-0936	CTTTTGGTACCCAGCCCGTACTCATGCTCG	construction of plasmid pKF551-1
KFO-0937	GTTTTTTTTAATACGACTCACTATAGGGAGGCA AGGACGAAACGAGCC	amplification of CrfA DNA template for T7 *in vitro* transcription
KFO-0938	ACACCAAACCCGCCGCGG	amplification of CrfA DNA template for T7 *in vitro* transcription
KFO-0966	CTTCTTTTCGGCGGACATAC	primer extension *hfq-hflX* mRNA
KFO-0983	GAATTGGCGATAGACCATGAAGAAGCTC	construction of plasmid pKF563-1
KFO-0984	GTCTATCGCCAATTCCTGCGACGTCT	construction of plasmid pKF563-1

KFO-0989	AATGAGGCGTTCCTTCAGATGAACATG	construction of plasmid pKF566-1
KFO-0990	AAGGAACGCCTCATTCATCAACACGC	construction of plasmid pKF566-1
KFO-1298	TCTCTGGTGGGCTCGTTTCGTCCTTGCAGT	oligo probe for CrfA
KFO-1575	CATCTGGGCGCATGCCGCCGCCGGGAG	construction of plasmid pKF732-3
KFO-1576	CGGCGGCATGCGCCCAGATGCTCGACGC	construction of plasmid pKF732-3
KFO-1607	GATATCTGGATCCACGAATTCGAGAGCTGCAT GCCGATATAGG	construction of plasmid pKF754-15
KFO-1610	AGACGCGTCACGGCCGAAGTCGACATGCAGC CAGCCG	construction of plasmid pKF754-15
KFO-1611	GATATCTGGATCCACGAATTCGTGTCGGCGCG CATGATGAAGA	construction of plasmid pKF747-1
KFO-1614	AGACGCGTCACGGCCGAAGACATTGACGATG CGGCCGAAC	construction of plasmid pKF747-1
KFO-1641	GCCGGGGGCCGACGCAATTCCTTCACGAC	construction of plasmid pKF740-1
KFO-1642	AATTGCGTCGGCCCCCGGCGGACAGC	construction of plasmid pKF740-1
KFO-1654	AAGCGTTCACTCGGCCGCCATCAGCCTTACCC C	construction of plasmid pKF754-15
KFO-1655	GGGGTAAGGCTGATGGCGGCCGAGTGAACGC TT	construction of plasmid pKF754-15
KFO-1656	GCTGGAGACGAGCACCTCAACCCGGGAACGT AAGG	construction of plasmid pKF747-1
KFO-1657	CGTTCCCGGGTTGAGGTGCTCGTCTCCAGCGA AG	construction of plasmid pKF747-1
KFO-1684	GATATCTGGATCCACGAATTCGCCGGAGCCAG TCAAGCGA	construction of plasmid pKF766-8
KFO-1685	CATCGAGCGCTGGGTTGCAGGGCGCCTTCA	construction of plasmid pKF766-8
KFO-1686	CGCCCTGCAACCCAGCGCTCGATGCTCGGA	construction of plasmid pKF766-8
KFO-1687	AGACGCGTCACGGCCGAAGAGCGCCGACCGT CTCCTGA	construction of plasmid pKF766-8
KFO-1785	GTTTTCATATGAGCCATATTCAACGGG	construction of plasmid pKF776-1
KFO-1786	GTTTTGTCGACTTAGAAAAACTCATCGAGCATC	construction of plasmid pKF776-1
KFO-2067	TACAGTTTACGAACCGAACAGGC	transposon-specific primer for mapping of MAR2xT7 insertion sites; PMFLGM.GB-3a primer (16)
KFO-2069	TGTCAACTGGGTTCGTGCCTTCATCCG	nested transposon-specific primer for mapping of MAR2xT7 insertion sites; PMFLGM.GB-2a primer (16)
KFO-2071	GACCGAGATAGGGTTGAGTG	sequencing of PCR products for mapping of MAR2xT7 insertion sites; PMFLGM.GB-4a primer (16)
KFO-2481	GATATCTGGATCCACGAATTCGCCTTCTCGATT AACGTCGGC	construction of plasmid pKF889-5
KFO-2482	TTGTAGTCGATGTCGTGGTCCTTGTAGTCGCC GTCGTGGTCCTTGTAGTCGAAGCGCGCGTGG ATCGT	construction of plasmid pKF889-5
KFO-2483	ACTACAAGGACCACGACATCGACTACAAGGAC GACGACGACAAGTAGTAAGCGTCGGGATCCAA GACC	construction of plasmid pKF889-5
KFO-2484	AGACGCGTCACGGCCGAAGCCAGCCGTTGAA CTTGAAGC	construction of plasmid pKF889-5
KPO-1524	CAACGGGAATCCTGCTCTG	construction of plasmid pKF755-3
KPO-1528	CAGAGCAGGATTCCCGTTGAGCACCGCCAGG TGCG	construction of plasmid pKF755-3
M13fwd	GTAAAACGACGGCCAGT	sequencing of plasmids with backbone pBVMCS-6

Supplementary Table S5 – Plasmids

plasmid ID	Description	Backbone/marker	Reference
pBVMCS-6	empty vector	pBVMCS-6/CmR	(3)
pGFPC-2	empty vector	pGFPC-2/KanR	(3)
pXGFP-5	empty vector	pXGFP-5/TetR	(3)
pNPTS138	empty vector	pNPTS138/KanR	M. R. Alley, unpublished
pMAR2xT7	Himar1 transposon delivery vector	pMAR2xT7/GentR	(16)
pKF383-7	expression of *chvR::lacZ* transcriptional fusion (-109 to +9 of chvR relative to TSS); integration into *xyl* locus	pXGFP-5/TetR	(1)
pKF385-2	expression of *rsaA::gfp* translational fusion (up to +45 of *rsaA* relative to the translational start site) under control of the *rsaA* promoter; integration into *rsaA* locus	pGFPC-2/KanR	(1)
pKF482-1	expression of *rusT* under control of the *van* promotor	pBVMCS-6/KanR	this study
pKF493-2	expression of *CCNA_02895::gfp* translational fusion (-269 to +60 of *CCNA_02895* relative to the translational start site) under control of the *rsaA* promotor, integration into *rsaA* locus	pGFPC-2/KanR	this study
pKF494-2	expression of *CCNA_03263::gfp* translational fusion (-116 to +60 of *CCNA_03263* relative to the translational start site) under control of the *rsaA* promotor, integration into *rsaA* locus	pGFPC-2/KanR	this study
pKF495-1	expression of *CCNA_01042::gfp* translational fusion (-225 of *CCNA_01043* to +60 of *CCNA_01042* relative to the translational start site) under control of the *rsaA* promotor, integration into *rsaA* locus	pGFPC-2/KanR	this study
pKF496-2	expression of *CCNA_02400::gfp* translational fusion (-32 to +60 of *CCNA_02400* relative to the translational start site) under control of the *rsaA* promotor, integration into *rsaA* locus	pGFPC-2/KanR	this study
pKF497-2	expression of *ompW::gfp* translational fusion (-40 to +75 of *ompW* relative to the translational start site) under control of the *rsaA* promotor, integration into *rsaA* locus	pGFPC-2/KanR	this study
pKF498-1	expression of *CCNA_01738::gfp* translational fusion (-86 to +60 of *CCNA_01738* relative to the translational start site) under control of the *rsaA* promotor, integration into *rsaA* locus	pGFPC-2/KanR	this study
pKF499-1	expression of *CCNA_02380::gfp* translational fusion (-71 to +60 of *CCNA_02380* relative to the translational start site) under control of the *rsaA* promotor, integration into *rsaA* locus	pGFPC-2/KanR	this study
pKF500-2	expression of *CCNA_03181::gfp* translational fusion (-156 to +60 of *CCNA_03181* relative to the translational start site) under control of the *rsaA* promotor, integration into *rsaA* locus	pGFPC-2/KanR	this study
pKF501-1	expression of CCNA*_00210::gfp* translational fusion (-59 to +60 of *CCNA_00210* relative to the translational start site) under control of the *rsaA* promotor, integration into *rsaA* locus	pGFPC-2/KanR	this study
pKF502-1	expression of CCNA*_01043::g*fp translational fusion (-43 to +60 of *CCNA_01043* relative to the translational start site) under control of the *rsaA* promotor, integration into *rsaA* locus	pGFPC-2/KanR	this study
pKF503-2	expression of CCNA*_03248::gfp* translational fusion (-49 to +60 of *CCNA_03248* relative to the translational start site) under control of the *rsaA* promotor, integration into *rsaA* locus	pGFPC-2 /KanR	this study

pKF504-1	expression of *CCNA_03294::gfp* translational fusion (-96 to +60 of *CCNA_03294* relative to the translational start site) under control of the *rsaA* promotor, integration into *rsaA* locus	pGFPC-2/KanR	this study
pKF505-1	expression of *CCNA_01819::gfp* translational fusion (--179 to +60 of *CCNA_01819* relative to the translational start site) under control of the *rsaA* promotor, integration into *rsaA* locus	pGFPC-2/KanR	this study
pKF506-1	expression of *CCNA_01820::gfp* translational fusion (-53 to +60 of *CCNA_01819* relative to the translational start site) under control of the *rsaA* promotor, integration into *rsaA* locus	pGFPC-2/KanR	this study
pKF507-1	expression of *rusT::lacZ* transcriptionalfusion (-x to +x of *rusT* relative to TSS); integration into *xyl* locus	pXGFP-5/TetR	this study
pKF511-8	allelic replacement of *rusT*	pNPTS138/KanR	this study
pKF518-2	expression of *rusT-M1* (C53G) under control of the *van* promotor	pBVMCS-6/CmR	this study
pKF544-1	expression of *CCNA_02741::gfp* translational fusion (-99 to +75 of *CCNA_02741* relative to the translational start site) under control of the *rsaA* promotor, integration into *rsaA* locus	pGFPC-2/KanR	this study
pKF545-1	expression of *CCNA_03774::gfp* translational fusion (-29 to +60 of *CCNA_03774* relative to the translational start site) under control of the *rsaA* promotor, integration into *rsaA* locus	pGFPC-2/KanR	this study
pKF546-1	expression of *CCNA_00338::gfp* translational fusion (-94 to +60 of *CCNA_00338* relative to the translational start site) under control of the *rsaA* promotor, integration into *rsaA* locus	pGFPC-2/KanR	this study
pKF547-1	expression of *glnBA::gfp* translational fusion (-424 to +60 of *glnB* relative to the translational start site) under control of the *rsaA* promotor, integration into *rsaA* locus	pGFPC-2/KanR	this study
pKF548-1	expression of *glnB::gfp* translational fusion (-42 to +60 of *glnB* relative to the translational start site) under control of the *rsaA* promotor, integration into *rsaA* locus	pGFPC-2/KanR	this study
pKF549-1	expression of *CCNA_02342::gfp* translational fusion (-35 to +60 of *CCNA_02342* relative to the translational start site) under control of the *rsaA* promotor, integration into *rsaA* locus	pGFPC-2/KanR	this study
pKF550-3	expression of *CCNA_02379::gfp* translational fusion (-71 to +60 of *CCNA_02379* relative to the translational start site) under control of the *rsaA* promotor, integration into *rsaA* locus	pGFPC-2/KanR	this study
pKF551-1	expression of *CCNA_03360::gfp* translational fusion (-282 to +60 of *CCNA_03360* relative to the translational start site) under control of the *rsaA* promotor, integration into *rsaA* locus	pGFPC-2/KanR	this study
pKF563-1	expression of *ompW::gfp* translational fusion (SNE in *ompW* relative to the TSS) under control of the *rsaA* promotor, integration into *rsaA* locus	pGFPC-2/KanR	this study
pKF566-1	expression of *CCNA_00210::gfp* translational fusion (SNE in *CCNA_00210* relative to the TSS) under control of the *rsaA* promotor, integration into *rsaA* locus	pGFPC-2/KanR	this study
pKF732-3	expression of *rusT-SL3mut* under control of the *van* promotor	pBVMCS-6/CmR	
pKF740-1	expression of *CCNA_02895::gfp* translational fusion (SNE in *CCNA_02895* relative to the TSS) under control of the *rsaA* promotor, integration into *rsaA* locus	pGFPC-2/KanR	this study
pKF776-1	expression of *rusT::nptII* transcriptionalfusion (-x to +x of *rusT* relative to TSS); integration into *xyl* locus	pXGFP-5/TetR	this study
pKF889-5	insertion of C-terminal 3xFLAG in *CCNA_00210*	pNPTS138/KanR	this study

Supplementary Table S6 – Bacterial strains

strain	stock name	bacterium	genotype/relevant markers	source/reference
wild type	KFS-0006	*C. crescentus NA1000*		Laboratory stock
ΔvanAB	KFS-0058	*C. crescentus NA1000*	*ΔvanAB*	(1)
	KFS-0096	*C. crescentus NA1000*	*ΔvanAB* + pBVMCS-6	this study
3xFLAG::hfq	KFS-0344	*C. crescentus NA1000*	*3XFLAG::hfq*	(1)
Δhfq	KFS-0570	*C. crescentus NA1000*	*Δhfq::TetR*	(17)
	KFS-0830	*C. crescentus NA1000*	*ΔvanAB* + pKF482-1	this study
ΔvanAB ΔrusT	KFS-0849	*C. crescentus NA1000*	*ΔvanAB ΔrusT*	this study
	KFS-1200	*C. crescentus NA1000*	*ΔvanAB ΔrusT PrsaA:: pKF493-2* + pBVMCS-6	this study
	KFS-1201	*C. crescentus NA1000*	*ΔvanAB ΔrusT PrsaA:: pKF493-2* + pKF482-1	this study
	KFS-1202	*C. crescentus NA1000*	*ΔvanAB ΔrusT PrsaA:: pKF494-2* + pBVMCS-6	this study
	KFS-1203	*C. crescentus NA1000*	*ΔvanAB ΔrusT PrsaA:: pKF494-2* + pKF482-1	this study
	KFS-1204	*C. crescentus NA1000*	*ΔvanAB ΔrusT PrsaA:: pKF495-1* + pBVMCS-6	this study
	KFS-1205	*C. crescentus NA1000*	*ΔvanAB ΔrusT PrsaA:: pKF495-1* + pKF482-1	this study
	KFS-1206	*C. crescentus NA1000*	*ΔvanAB ΔrusT PrsaA::pKF496-2* + pBVMCS-6	this study
	KFS-1207	*C. crescentus NA1000*	*ΔvanAB ΔrusT PrsaA::pKF496-2* + pKF482-1	this study
	KFS-1209	*C. crescentus NA1000*	*ΔvanAB ΔrusT PrsaA::pKF497-2* + pBVMCS-6	this study
	KFS-1210	*C. crescentus NA1000*	*ΔvanAB ΔrusT PrsaA::pKF497-2* + pKF482-1	this study
	KFS-1211	*C. crescentus NA1000*	*ΔvanAB ΔrusT PrsaA::pKF497-2* + pKF518-2	this study
	KFS-1212	*C. crescentus NA1000*	*ΔvanAB ΔrusT PrsaA::pKF498-1* + pBVMCS-6	this study
	KFS-1213	*C. crescentus NA1000*	*ΔvanAB ΔrusT PrsaA::pKF498-1* + pKF482-1	this study
	KFS-1214	*C. crescentus NA1000*	*ΔvanAB ΔrusT PrsaA::pKF501-1* + pBVMCS-6	this study
	KFS-1215	*C. crescentus NA1000*	*ΔvanAB ΔrusT PrsaA::pKF501-1* + pKF482-1	this study

	KFS-1216	*C. crescentus NA1000*	Δ*vanAB* Δ*rusT PrsaA::pKF501-1* + pKF518-2	this study
	KFS-1217	*C. crescentus NA1000*	Δ*vanAB* Δ*rusT PrsaA::pKF502-1* + pBVMCS-6	this study
	KFS-1218	*C. crescentus NA1000*	Δ*vanAB* Δ*rusT PrsaA::pKF502-1* + pKF482-1	this study
	KFS-1219	*C. crescentus NA1000*	Δ*vanAB* Δ*rusT PrsaA::pKF503-2* + pBVMCS-6	this study
	KFS-1220	*C. crescentus NA1000*	Δ*vanAB* Δ*rusT PrsaA::pKF503-2* + pKF482-1	this study
	KFS-1221	*C. crescentus NA1000*	Δ*vanAB* Δ*rusT PrsaA::pKF504-1* + pBVMCS-6	this study
	KFS-1222	*C. crescentus NA1000*	Δ*vanAB* Δ*rusT PrsaA::pKF504-1* + pKF482-1	this study
	KFS-1224	*C. crescentus NA1000*	Δ*vanAB* Δ*rusT PrsaA::pKF505-1* + pBVMCS-6	this study
	KFS-1225	*C. crescentus NA1000*	Δ*vanAB* Δ*rusT PrsaA::pKF505-1* + pKF482-1	this study
	KFS-1227	*C. crescentus NA1000*	Δ*vanAB* Δ*rusT PrsaA::pKF506-1* + pBVMCS-6	this study
	KFS-1228	*C. crescentus NA1000*	Δ*vanAB* Δ*rusT PrsaA::pKF506-1* + pKF482-1	this study
	KFS-1229	*C. crescentus NA1000*	Δ*vanAB* Δ*rusT PrsaA::pKF544-1* + pBVMCS-6	this study
	KFS-1230	*C. crescentus NA1000*	Δ*vanAB* Δ*rusT PrsaA::pKF544-1* + pKF482-1	this study
	KFS-1231	*C. crescentus NA1000*	Δ*vanAB* Δ*rusT PrsaA::pKF545-1* + pBVMCS-6	this study
	KFS-1232	*C. crescentus NA1000*	Δ*vanAB* Δ*rusT PrsaA::pKF545-1* + pKF482-1	this study
	KFS-1234	*C. crescentus NA1000*	Δ*vanAB* Δ*rusT PrsaA::pKF546-1* + pBVMCS-6	this study
	KFS-1235	*C. crescentus NA1000*	Δ*vanAB* Δ*rusT PrsaA::pKF546-1* + pKF482-1	this study
	KFS-1236	*C. crescentus NA1000*	Δ*vanAB* Δ*rusT PrsaA::pKF547-1* + pBVMCS-6	this study
	KFS-1237	*C. crescentus NA1000*	Δ*vanAB* Δ*rusT PrsaA::pKF547-1* + pKF482-1	this study
	KFS-1238	*C. crescentus NA1000*	Δ*vanAB* Δ*rusT PrsaA::pKF548-1* + pBVMCS-6	this study
	KFS-1239	*C. crescentus NA1000*	Δ*vanAB* Δ*rusT PrsaA::pKF548-1* + pKF482-1	this study
	KFS-1240	*C. crescentus NA1000*	Δ*vanAB* Δ*rusT PrsaA::pKF549-1* + pBVMCS-6	this study
	KFS-1241	*C. crescentus NA1000*	Δ*vanAB* Δ*rusT PrsaA::pKF549-1* + pKF482-1	this study
	KFS-1242	*C. crescentus NA1000*	Δ*vanAB* Δ*rusT PrsaA::pKF550-3* + pBVMCS-6	this study
	KFS-1243	*C. crescentus NA1000*	Δ*vanAB* Δ*rusT PrsaA::pKF550-3* + pKF482-1	this study

	KFS-1244	*C. crescentus NA1000*	Δ*vanAB* Δ*rusT PrsaA::pKF551-1* + pBVMCS-6	this study
	KFS-1245	*C. crescentus NA1000*	Δ*vanAB* Δ*rusT PrsaA::pKF551-1* + pKF482-1	this study
	KFS-1246	*C. crescentus NA1000*	Δ*vanAB* Δ*rusT PrsaA::pKF563-1* + pBVMCS-6	this study
	KFS-1247	*C. crescentus NA1000*	Δ*vanAB* Δ*rusT PrsaA::pKF563-1* + pKF482-1	this study
	KFS-1248	*C. crescentus NA1000*	Δ*vanAB* Δ*rusT PrsaA::pKF563-1* + pKF518-2	this study
	KFS-1255	*C. crescentus NA1000*	Δ*vanAB* Δ*rusT PrsaA::pKF566-1* + pBVMCS-6	this study
	KFS-1256	*C. crescentus NA1000*	Δ*vanAB* Δ*rusT PrsaA::pKF566-1* + pKF482-1	this study
	KFS-1257	*C. crescentus NA1000*	Δ*vanAB* Δ*rusT PrsaA::pKF566-1* + pKF518-2	this study
	KFS-1261	*C. crescentus NA1000*	Δ*vanAB* Δ*rusT PrsaA::pKF358-2* + pBVMCS-6	this study
	KFS-1262	*C. crescentus NA1000*	Δ*vanAB* Δ*rusT PrsaA::pKF358-2* + pKF482-1	this study
	KFS-1263	*C. crescentus NA1000*	Δ*vanAB* Δ*rusT PrsaA::pKF358-2* + pKF518-2	this study
	KFS-1283	*C. crescentus NA1000*	Δ*vanAB* Δ*rusT PrsaA::pKF499-1* + pBVMCS-6	this study
	KFS-1284	*C. crescentus NA1000*	Δ*vanAB* Δ*rusT PrsaA::pKF499-1* + pKF482-1	this study
	KFS-1285	*C. crescentus NA1000*	Δ*vanAB* Δ*rusT PrsaA::pKF500-2* + pBVMCS-6	this study
	KFS-1286	*C. crescentus NA1000*	Δ*vanAB* Δ*rusT PrsaA::pKF500-2* + pKF482-1	this study
	KFS-1287	*C. crescentus NA1000*	Δ*vanAB* Δ*rusT* + pBVMCS-6	this study
	KFS-1288	*C. crescentus NA1000*	Δ*vanAB* Δ*rus*T + pKF482-1	this study
	KFS-1289	*C. crescentus NA1000*	Δ*vanAB* Δ*rusT* + pKF518-2	this study
	KFS-1290	*C. crescentus NA1000*	Δ*vanAB* Δ*rusT* Δ*hfq::TetR* + pBVMCS-6	this study
	KFS-1291	*C. crescentus NA1000*	Δ*vanAB* Δ*rusT* Δ*hfq::TetR* + pKF482-1	this study
	KFS-1292	*C. crescentus NA1000*	Δ*vanAB* Δ*rusT* Δ*hfq::TetR PrsaA::pKF385-2* + pBVMCS-6	this study
	KFS-1293	*C. crescentus NA1000*	Δ*vanAB* Δ*rusT* Δ*hfq::TetR PrsaA::pKF385-2* + pKF482-1	this study
	KFS-1294	*C. crescentus NA1000*	Δ*vanAB* Δ*rusT* Δ*hfq::TetR PrsaA:: pKF493-2* + pBVMCS-6	this study
	KFS-1295	*C. crescentus NA1000*	Δ*vanAB* Δ*rusT* Δ*hfq::TetR PrsaA:: pKF493-2* + pKF482-1	this study
	KFS-1296	*C. crescentus NA1000*	Δ*vanAB* Δ*rusT* Δ*hfq::TetR PrsaA:: pKF494-2* + pBVMCS-6	this study

	KFS-1297	*C. crescentus NA1000*	*ΔvanAB ΔrusT Δhfq::TetR PrsaA:: pKF494-2* + pKF482-1	this study
	KFS-1298	*C. crescentus NA1000*	*ΔvanAB ΔrusT Δhfq::TetR PrsaA:: pKF495-1* + pBVMCS-6	this study
	KFS-1299	*C. crescentus NA1000*	*ΔvanAB ΔrusT Δhfq::TetR PrsaA:: pKF495-1* + pKF482-1	this study
	KFS-1300	*C. crescentus NA1000*	*ΔvanAB ΔrusT Δhfq::TetR PrsaA:: pKF501-1* + pBVMCS-6	this study
	KFS-1301	*C. crescentus NA1000*	*ΔvanAB ΔrusT Δhfq::TetR PrsaA:: pKF501-1* + pKF482-1	this study
	KFS-1302	*C. crescentus NA1000*	*ΔvanAB ΔrusT Δhfq::TetR PrsaA:: pKF503-2* + pBVMCS-6	this study
	KFS-1303	*C. crescentus NA1000*	*ΔvanAB ΔrusT Δhfq::TetR PrsaA:: pKF503-2* + pKF482-1	this study
ΔvanAB ΔrusT Δhfq	KFS-1648	*C. crescentus NA1000*	*ΔvanAB ΔrusT Δhfq::TetR*	this study
	KFS-1678	*C. crescentus NA1000*	*ΔvanAB ΔrusT PrsaA::pKF566-1* + pBVMCS-6	this study
	KFS-1679	*C. crescentus NA1000*	*ΔvanAB ΔrusT PrsaA::pKF566-1* + pKF482-1	this study
	KFS-1680	*C. crescentus NA1000*	*ΔvanAB ΔrusT PrsaA::pKF566-1* + pKF518-2	this study
	KFS-1681	*C. crescentus NA1000*	*ΔvanAB ΔrusT PrsaA::pKF740-1* + pBVMCS-6	this study
	KFS-1682	*C. crescentus NA1000*	*ΔvanAB ΔrusT PrsaA::pKF740-1* + pKF482-1	this study
	KFS-1683	*C. crescentus NA1000*	*ΔvanAB ΔrusT PrsaA::pKF740-1* + pKF518-2	this study
ΔvanAB CCNA_00210::3xFLAG	KFS-2117	*C. crescentus NA1000*	*ΔvanAB CCNA_00210::3xFLAG*	this study
	KFS-2134	*C. crescentus NA1000*	*ΔvanAB CCNA_00210::3xFLAG* + pBVMCS-6	this study
	KFS-2135	*C. crescentus NA1000*	*ΔvanAB CCNA_00210::3xFLAG* + pKF482-1	this study
ΔvanAB ΔrusT CCNA_00210::3xFLAG	KFS-2173	*C. crescentus NA1000*	*ΔvanAB ΔrusT CCNA_00210::3xFLAG*	this study
	KFS-2219	*C. crescentus NA1000*	*ΔvanAB ΔrusT CCNA_00210::3xFLAG* + pBVMCS-6	this study
	KFS-2220	*C. crescentus NA1000*	*ΔvanAB ΔrusT CCNA_00210::3xFLAG* + pKF482-1	this study
ntrX-HA	FC3561	*C. crescentus* CB15	*ntrX::ntrX-HA*	(18)
E. coli TOP10	KFS-0088	*E. coli*	*F- mcrA Δ(mrr-hsdRMS-mcrBC) Φ80lacZΔM15 ΔlacX74 recA1 araD139 Δ(ara-leu)7697 galU galK rpsL endA1 nupG λ-*	invitrogen
E. coli WM3064		*E. coli*	*thrB*1004 *pro thi rpsL hsdS lacZ*ΔM15 RP4-1360 Δ(*araBAD)567 ΔdapA*1341::[*erm pir*(wt)]	(6)

SUPPORTING REFERENCES

1. Fröhlich KS, Förstner KU, & Gitai Z (2018) Post-transcriptional gene regulation by an Hfq-independent small RNA in Caulobacter crescentus. *Nucleic Acids Res* 46(20):10969-10982.
2. Larsen RA, Wilson MM, Guss AM, & Metcalf WW (2002) Genetic analysis of pigment biosynthesis in *Xanthobacter autotrophicus* Py2 using a new, highly efficient transposon mutagenesis system that is functional in a wide variety of bacteria. *Arch Microbiol* 178(3):193-201.
3. Thanbichler M, Iniesta AA, & Shapiro L (2007) A comprehensive set of plasmids for vanillate- and xylose-inducible gene expression in *Caulobacter crescentus*. *Nucleic Acids Res* 35(20):e137.
4. Skerker JM, Prasol MS, Perchuk BS, Biondi EG, & Laub MT (2005) Two-component signal transduction pathways regulating growth and cell cycle progression in a bacterium: a system-level analysis. *PLoS Biol* 3(10):e334.
5. Ely B (1991) Genetics of *Caulobacter crescentus*. *Methods Enzymol* 204:372-384.
6. Saltikov CW & Newman DK (2003) Genetic identification of a respiratory arsenate reductase. *Proc Natl Acad Sci U S A* 100(19):10983-10988.
7. Chao Y, Papenfort K, Reinhardt R, Sharma CM, & Vogel J (2012) An atlas of Hfq-bound transcripts reveals 3' UTRs as a genomic reservoir of regulatory small RNAs. *EMBO J* 31(20):4005-4019.
8. Edgar R, Domrachev M, & Lash AE (2002) Gene Expression Omnibus: NCBI gene expression and hybridization array data repository. *Nucleic Acids Res* 30(1):207-210.
9. Huber M, Fröhlich KS, Radmer J, & Papenfort K (2020) Switching fatty acid metabolism by an RNA-controlled feed forward loop. *Proc Natl Acad Sci U S A* 117(14):8044-8054.
10. Papenfort K*, et al.* (2006) σ^E-dependent small RNAs of *Salmonella* respond to membrane stress by accelerating global *omp* mRNA decay. *Mol Microbiol* 62(6):1674-1688.
11. Fröhlich KS, Haneke K, Papenfort K, & Vogel J (2016) The target spectrum of SdsR small RNA in *Salmonella*. *Nucleic Acids Res* 44(21):10406-10422.
12. Fröhlich KS, Papenfort K, Fekete A, & Vogel J (2013) A small RNA activates CFA synthase by isoform-specific mRNA stabilization. *EMBO J* 32(22):2963-2979.
13. Corpet F (1988) Multiple sequence alignment with hierarchical clustering. *Nucleic Acids Res* 16(22):10881-10890.
14. Rehmsmeier M, Steffen P, Hochsmann M, & Giegerich R (2004) Fast and effective prediction of microRNA/target duplexes. *RNA* 10(10):1507-1517.
15. Mann M, Wright PR, & Backofen R (2017) IntaRNA 2.0: enhanced and customizable prediction of RNA-RNA interactions. *Nucleic Acids Res* 45(W1):W435-W439.
16. Liberati NT*, et al.* (2006) An ordered, nonredundant library of Pseudomonas aeruginosa strain PA14 transposon insertion mutants. *Proc Natl Acad Sci U S A* 103(8):2833-2838.
17. Irnov I*, et al.* (2017) Crosstalk between the tricarboxylic acid cycle and peptidoglycan synthesis in Caulobacter crescentus through the homeostatic control of alpha-ketoglutarate. *PLoS Genet* 13(8):e1006978.
18. Stein BJ, Fiebig A, & Crosson S (2021) The ChvG-ChvI and NtrY-NtrX Two-Component Systems Coordinately Regulate Growth of Caulobacter crescentus. *J Bacteriol* 203(17):e0019921.
